# Antiphospholipid antibodies in acute and post-treatment Lyme disease

**DOI:** 10.1128/iai.00192-26

**Published:** 2026-06-30

**Authors:** Muskan Shrestha, Anna F. Hickman, Yuelin Zhong, Audre Zvinys, Suzanne D. Vernon, John B. Miller, Alison W. Rebman, John N. Aucott, Elizabeth J. Horn, Linden T. Hu, Peter J. Gwynne

**Affiliations:** 1 Tufts Graduate School of Biomedical Sciences & Tufts Lyme Disease Initiative, Boston, Massachusetts, USA; 2 Bateman Horne Center 114361, Murray, Utah, USA; 3 Lyme Disease Research Center, Division of Rheumatology, Johns Hopkins University School of Medicine 1500 , Baltimore, Maryland, USA; 4 Lyme Disease Biobank, Portland, Oregon, USA; Tulane University, New Orleans, Louisiana, USA

**Keywords:** autoantibodies, Infection-associated chronic illness, Autoimmunity, Lyme disease

## Abstract

Acute Lyme disease is caused by a *Borrelia* infection and typically responds to antibiotic treatment, but post-infectious chronic symptoms are common. The precise origin of these post-treatment symptoms is unknown; dysregulation of immune responses raised during the initial infection is likely to contribute. Antilipid antibodies are associated with several autoimmune diseases and have been shown to arise in both acute and post-treatment Lyme disease. To assess the potential contribution of antilipid antibodies to Lyme disease pathology and their possible use as biomarkers of both acute and chronic disease, we performed a survey of patient sera for antilipid antibodies during and after *Borrelia burgdorferi* infection. Results were similar in cross-sectional and longitudinal samples drawn from two different biobanks. Three antiphospholipid antibodies were elevated during infection, with two (antiphosphatidic acid and antiphosphatidylserine) significantly elevated even at the day of diagnosis before seroconversion on conventional tests. A subset of patients with chronic symptoms is identifiable by persistent elevation in antiphosphatidylserine; these antibodies found in post-treatment Lyme disease were not found in a panel of sera from patients with look-alike autoimmune disorders. Persistent elevation in antiphosphatidylserine may drive autoimmune-like symptoms of Lyme disease in some patients and could serve as a biomarker for chronic disease. The detection of antiphospholipid antibodies induced early in infection could also improve the diagnosis of acute infections.

## INTRODUCTION

Lyme disease is the most common vector-borne infection in North America and Europe and is caused in the United States mainly by the spirochete *Borrelia burgdorferi*. Early infection typically manifests within days to weeks with symptoms such as fatigue, fever, headache, myalgias, arthralgias, and the hallmark erythema migrans rash (1). If left untreated, *B. burgdorferi* can disseminate and lead to cardiac involvement, arthritis, and neurologic complications. Most patients are treated with a 2- to 3-week course of oral antibiotics. Although antibiotic therapy is generally effective in clearing acute infection, approximately 10%–20% of treated individuals develop persistent, recurrent, and non-specific symptoms, referred to as post-treatment Lyme disease (PTLD) (2–4). The biological mechanisms underlying PTLD remain poorly defined, and diagnosis is hindered by the absence of objective biomarkers capable of distinguishing active disease, post-infectious persistent disease, or unrelated pathology. The existing molecular tests for acute, untreated disease rely on serological detection of anti-*Borrelia* protein antibodies, which often remain positive long after microbial clearance (5) and therefore provide limited insight into persistent symptoms or treatment response. Furthermore, many cases diagnosed clinically (based on risk factors and symptoms, including the erythema migrans) fail to seroconvert due to early treatment of infection (6, 7).

Autoimmune-like sequelae are known to follow numerous bacterial and viral infections, often with cryptic and overlapping symptom profiles (8). Emerging evidence suggests possible autoimmune origins of persistent symptoms in a subset of individuals; numerous autoantibodies have been found to be elevated in Lyme disease and particularly post-infectious Lyme arthritis patients (9–12). Dysregulation of proinflammatory cytokines such as CXCL13 (13) and IFN-γ (14) has also been associated with post-treatment symptoms. Meanwhile, elevated antilipid antibodies have been described in a number of autoimmune diseases, including systemic lupus erythematosus (15, 16), multiple sclerosis (17, 18), and the eponymous antiphospholipid syndrome (19, 20). The presence of antilipid autoantibodies represents a plausible but underexplored mechanism for the pathology of persistent symptoms in Lyme disease.

Unlike most bacteria, *B. burgdorferi* lacks many pathways required for the biosynthesis of membrane precursor fatty acids, cholesterol, and choline (21, 22). Lipid production instead depends on scavenging host-derived lipids for membrane biogenesis. The organism incorporates a diverse array of host phospholipids, including phosphatidylserine (PS), phosphatidic acid (PA), and phosphatidylethanolamine (PE), into its outer membrane, in addition to synthesizing a limited set of endogenous lipids such as phosphatidylcholine (PC), phosphatidylglycerol (PG), and galactosylated glycolipids (22, 23). This extensive lipid scavenging creates a unique immunologic interface in which self-lipids may be presented in the immunogenic context of a bacterial infection and may drive autoreactive immune responses.

Although most characterized immune responses to *B. burgdorferi* target protein antigens, infection also induces antibody responses against lipid antigens. Antibodies against galactosyl cholesterol and galactosyl diacylglycerol have been detected and persist following treatment in Lyme disease patients (24). These antibodies cross-react with host gangliosides, which have been proposed as a potential contributor to neurologic manifestations of infection (25). In addition to their role in humoral immunity, lipid antigens can activate innate immune pathways; for example, galactosyl diacylglycerol from *B. burgdorferi* stimulates natural killer T cells (26, 27), promoting the production of proinflammatory cytokines such as IFN-γ and facilitating downstream macrophage activation and bacterial clearance (28). Building on these observations, our prior work demonstrated that *B. burgdorferi* infection elicits robust antiphospholipid antibody responses in both murine models and human patients. These antibodies arise early during infection, target multiple host-derived lipid species incorporated into the bacterial membrane, and decline following antibiotic treatment (22).

There is a significant clinical need for the development of improved diagnostic tests for Lyme disease. The standard or modified two-tier test for acute disease uses an initial enzyme-linked immunosorbance assay (ELISA) followed by confirmatory immunoblotting or a second ELISA (29). While standard testing performs well in established infections, it has important limitations: sensitivity is poor in the first 2–3 weeks of infection (6, 30), and antibody responses often persist for months or years after successful treatment (5), limiting its utility for assessing ongoing infection or therapeutic response. Consequently, existing tests cannot reliably distinguish between active infection, post-treatment sequelae, or prior exposure.

Antiphospholipid antibodies are a potential contributor to the unexplained pathology of post-treatment Lyme disease. Their use as an adjunct diagnostic test in syphilis suggests that they could also offer a novel approach to assessing disease activity beyond conventional serological tests in Lyme disease. In this study, we build on prior work to evaluate the incidence and kinetics of antilipid antibodies in acute and post-treatment Lyme disease, with the goal of improving diagnostic accuracy and advancing understanding of the immunologic processes underlying persistent symptoms.

## MATERIALS AND METHODS

### Patient samples

Serum samples from the Johns Hopkins Lyme Disease Research Center were collected from participants enrolled in an ongoing longitudinal cohort study in the Mid-Atlantic United States, as previously described (31). At the baseline visit, participants were required to have a physician-diagnosed erythema migrans rash ≥5 cm present and to be antibiotic naïve or have started doxycycline treatment less than the previous week. Participants were also excluded for a range of co-morbidities, including pregnancy, autoimmune disease, HIV, hepatitis or other liver disease, cancer, or malignancy in the past 2 years, ME/CFS, fibromyalgia, chronic pain disorders, sleep apnea, chronic neurologic disease, current alcohol or drug abuse, or major psychiatric conditions. Participants are then seen at standardized post-treatment follow-up intervals. The current study tested samples from baseline and approximately 2, 6, and 12 months after completion of standard antibiotic therapy for acute Lyme disease. At both the 6-month and 1-year time points, participants were characterized as return to health (RTH), symptoms only, or PTLD based on the presence or absence of (i) symptoms (fatigue, musculoskeletal pain, and/or cognitive complaints) and (ii) functional impact, as measured by SF-36 version 2 (32), as previously described (33). In the current study, PTLD participants were selected for analysis if they met the criteria (symptoms and functional impact) at either 6 or 12 months, and RTH participants were selected if they met the criteria (neither symptoms nor functional impact) at both 6 and 12 months. In the PTLD group, the most common reported symptoms were fatigue (15/25), joint pain (12/25), and sleep disruption (12/25). Complete symptom data are available in Tables S1 to S5.

Lyme Disease Biobank serum samples were collected from participants enrolled with signs and symptoms of early Lyme disease on the East Coast and in the Upper Midwest of the United States (34). Endemic control samples were collected from individuals in the same regions without a history of Lyme disease or other tick-borne infections. Participants enrolled as cases provided an optional convalescent (second-draw sample) ~3 months after the initial draw. Serological testing, including a first-tier ELISA and IgM and IgG immunoblots, was performed on all samples as previously described (34, 35). Clinical data were collected at both blood draws, including information about constitutional symptoms. Samples were also collected from participants living on the West Coast of the United States who had a history of Lyme disease for at least 1 year and continued to have persistent symptoms (fatigue, musculoskeletal pain, neuropathy, migratory arthralgias, and/or cognitive impairment) for the past 6 months or more (>6 months after treatment). In this PTLD group, the most common symptoms were sleep impairment (38/48), joint pain (37/48), and musculoskeletal pain (36/48). Complete symptom data are available in Tables S1 to S5.

A small number of sera from a biobank at Tufts University were used to supplement the 3-month time point. These samples were collected from diagnostic discards and included patients between 60 and 100 days after an initial Lyme disease diagnosis made by either positive serology or presence of an erythema migrans.

All patients met the clinical diagnostic criteria recommended by the National Academy of Medicine (36). Long COVID patients were between the ages of 18 and 65 and met the clinical case definition for post-COVID-19 condition (37). Samples from BHC were EDTA plasma and included matched plasma controls. Blood was collected by venipuncture into EDTA tubes, processed immediately to collect plasma, and stored at −80°C.

Non-endemic controls were purchased from a commercial vendor (Pel-Freez) collected in Memphis, TN. Autoimmune disease controls were also purchased from commercial vendors (LGC Clinical Diagnostics and Sanguine Bio). The demographic details of Lyme and control sera are described in a Tables S1 to S5. Details of patient groups are described in Table 1.

**TABLE 1:** Patient samples used and their sources^*a*^

Group	Source (number)	Duration of disease	Method of diagnosis	Collection type	Figures
Endemic controls	LDB (48)	N/A	N/A	Cross-sectional	1 to 7A
Acute	LDB (48)	Day of diagnosis	Clinical or serological	Cross-sectional	4 and 6
3 months	LDB (40)	65–189 days from diagnosis	Clinical or serological	24 cross-sectional and 24 paired acute samples	1, 2, 5, and 6
	Tufts (8)				
>1 year PTLD	LDB (48)	1–40 years from initial diagnosis	Clinical or serological Lyme, self-reported PTLD	Cross-sectional	1, 2, 5, 6, and 7A
Day 0 (RTH at 6 months and 1 year)	JHU (29)	Day of diagnosis	Clinical or serological	Longitudinal	3, 4, and 6
3 months (RTH at 6 months and 1 year)	JHU (29)	2 months from end of treatment	Clinical or serological	Longitudinal	4 and 6
6 months (RTH at 6 months and 1 year)	JHU (29)	6 months from end of treatment	Clinical or serological	Longitudinal	4 and 6
1 year RTH	JHU (26)	1 year from end of treatment	Clinical or serological Lyme, RTH by 1 year symptoms	Longitudinal	4 and 6
Day 0 (PTLD at 6 months or 1 year)	JHU (26)	Day of diagnosis	Clinical or serological	Longitudinal	3, 4, and 6
3 months (PTLD at 6 months or 1 year)	JHU (24)	2 months from end of treatment	Clinical or serological	Longitudinal	4 and 6
6 months (PTLD at 6 months or 1 year)	JHU (26)	6 months from end of treatment	Clinical or serological Lyme, PTLD by clinical presentation	Longitudinal	4 and 6
1 year (PTLD at 6 months or 1 year)	JHU (25)	1 year from end of treatment	Clinical or serological Lyme, PTLD by clinical presentation	Longitudinal	4 and 6
Control plasma	BHC (10)	N/A	N/A	Cross-sectional	7B
ME/CFS plasma	BHC (10)	N/A	N/A	Cross-sectional	7B
Long COVID plasma	BHC (10)	N/A	N/A	Cross-sectional	7B
Non-endemic controls	Pel-freez (45)	N/A	N/A	Cross-sectional	1, 3, 4, 6, and 7A
Fibromyalgia	Sanguine (15)	N/A	N/A	Cross-sectional	7A
Systemic lupus erythematosus	LGC (10), sanguine (5)	N/A	N/A	Cross-sectional	7A
Multiple sclerosis		N/A	N/A	Cross-sectional	7A
Rheumatoid arthritis	LGC (10)	N/A	N/A	Cross-sectional	7A

^*a*^ BHC, Bateman Horne Center; JHU, Johns Hopkins University; LDB, Lyme Disease Biobank; ME/CFS, myalgic encephalomyelitis/chronic fatigue syndrome; PTLD, post-treatment Lyme disease. RTH, return to health.

### Enzyme-linked immunosorbance assay

Immulon-1B treated 96-well plates were coated with 2 µg lipid in 100 µL absolute ethanol. Lipids analyzed were PA, PC, PE, PG, PS, cardiolipin (CL), and galactosylcholesterol. All were sourced from Avanti Polar Lipids, with all phospholipids in the palmitoyl-oleoyl (16:0/18:1) form. The galactosyl cholesterol used was the palmitoylated form named previously as BbGL-1 (38). Plates were left at room temperature under a flow of air until ethanol had evaporated (1–2 hours), after which 150 µL 1% BSA (Fraction V, Fisher) in PBS was added to each well. Plates were blocked overnight at 4°C. Blocking buffer was removed using three washes of 200 µL PBS (using a Biotek 50TS automated microplate washer). The serum was diluted into the blocking buffer: seven twofold dilutions of serum were added for each sample, with 12 blocking buffer-only controls per plate. Typical dilution schemes ran from 1:100 to 1:6,400, with high- or low-titer samples repeated starting at dilutions up to 1:1,600 or as low as 1:25. The serum was incubated at room temperature for 3 hours to allow antibody adsorption, then removed using five washes of 200 µL PBS 0.1% Tween-80. A goat antihuman IgG horseradish peroxidase (HRP) conjugate (heavy + light chains, Thermo A18805) was added at a dilution of 1:4,000 in blocking buffer and incubated at room temperature for 1 hour before being removed using five washes of 200 µL PBS 0.1% Tween-80. Plates were developed with 100 µL tetramethylbenzidine HRP substrate (seracare SureBlue); the reaction was stopped after around 5 minutes with 100 µL 1 M HCl.

### Calculation of titers and statistical analysis

Absorbance was read at 450 nm using a Biotek Epoch 2 plate reader. A linear regression model was used to find titers using Microsoft Excel with the analytic solver add-in. A linear regression line was drawn through at least three linear points of the plot of OD_450_ against dilution (linearity defined at an *r*^2^ of >0.85). The cutoff value was derived from the 12 serum-free controls + 2.291 standard deviations, which gave a 97.5% confidence level (39). The intercept titer was the intercept of the regression line with the cutoff value. Samples without at least three linear points above 1.5× the cutoff value were deemed below the limit of detection and assigned a titer of 65 for graphing and statistical tests. This method effectively reduces threshold effects by calculating titers based on the overall shape of the dilution:signal curve rather than a single point. As the binary dilution series produced non-parametric data and all analyses included more than two groups, groups were compared using Kruskal-Wallis tests followed by Dunn’s multiple comparison tests. These analyses were performed in GraphPad Prism 11.0, which was also used to plot graphs.

## RESULTS

### Antiphospholipid IgG in post-treatment Lyme disease

**Fig 1:**
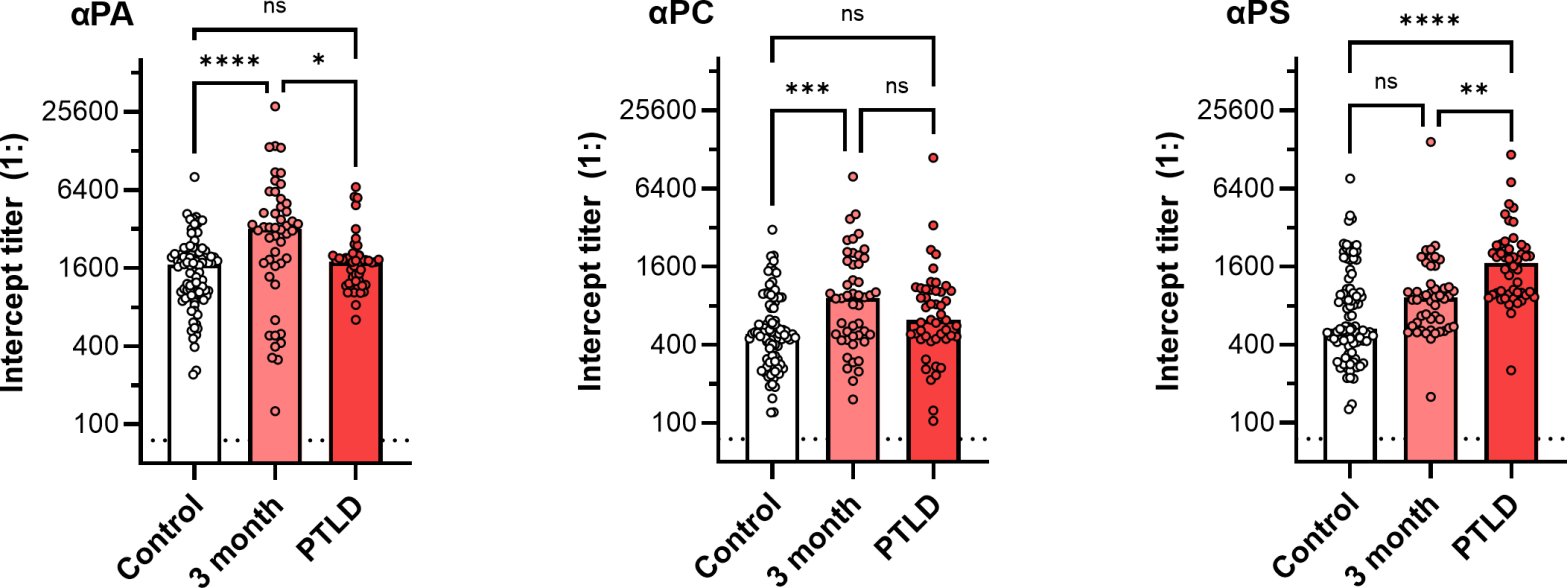
Titers of three IgG antiphospholipids in Lyme disease. Antiphosphatidic acid (αPA), antiphosphatidylcholine (αPC), and antiphosphatidylserine (αPS) were measured at two Lyme disease time points and in healthy controls. Lyme samples (sourced from Tufts and the LDB) were drawn from patients either 3 months after diagnosis regardless of symptoms or >1 year from diagnosis with persistent symptoms. Controls were from individuals in endemic areas (from the LDB) and non-endemic areas (Pel-Freez). *n* = 93 controls, 48 3 months, and 48 PTLD. Points are titers from individual patients; bars show the median. Pairwise comparisons were performed using Kruskal-Wallis tests with Dunn’s post hoc. *P < 0.05, **P < 0.01, ***P << 0.001, ****P < 0.0001. ns, not significant.

Our previous description of antiphospholipids in small numbers of Lyme disease patients (22) was replicated in two independent larger groups of 48 patients ~3 months (range 65–189 days, mean 102) after initial diagnosis and 48 patients >1 year after diagnosis and first treatment (range 1–40 years, mean 17.6) (Fig. 1). The latter group suffered persistent symptoms after treatment. Ninety-three controls were also analyzed: 48 endemic controls from the Lyme Disease Biobank (LDB) and 45 non-endemic controls from a commercial vendor. Two IgGs, antiphosphatidic acid (αPA) and antiphosphatidylcholine (αPC), were elevated at three months. This may reflect generally increasing antibody production after *B. burgdorferi* infection: circulating IgG peaks around 14 weeks in mice (40). Much of this increase is expected to be pathogen-specific IgG, but the parallel induction of autoantibodies may contribute to the pathology of disease in some patients. While αPA and αPC antibodies were similar to baseline controls in PTLD patients, antiphosphatidylserine (αPS) was significantly elevated. This suggests that the antiphospholipid antibodies are differently regulated, with αPA and αPC associated with acute disease and αPS with PTLD. All groups had significant variability between individuals: endemic controls in particular may have had prior exposure. The number of available PTLD samples and the heterogeneity in reported symptoms mean that this study was not powered to test correlations between antilipid antibodies and specific symptoms.

**Fig 2:**
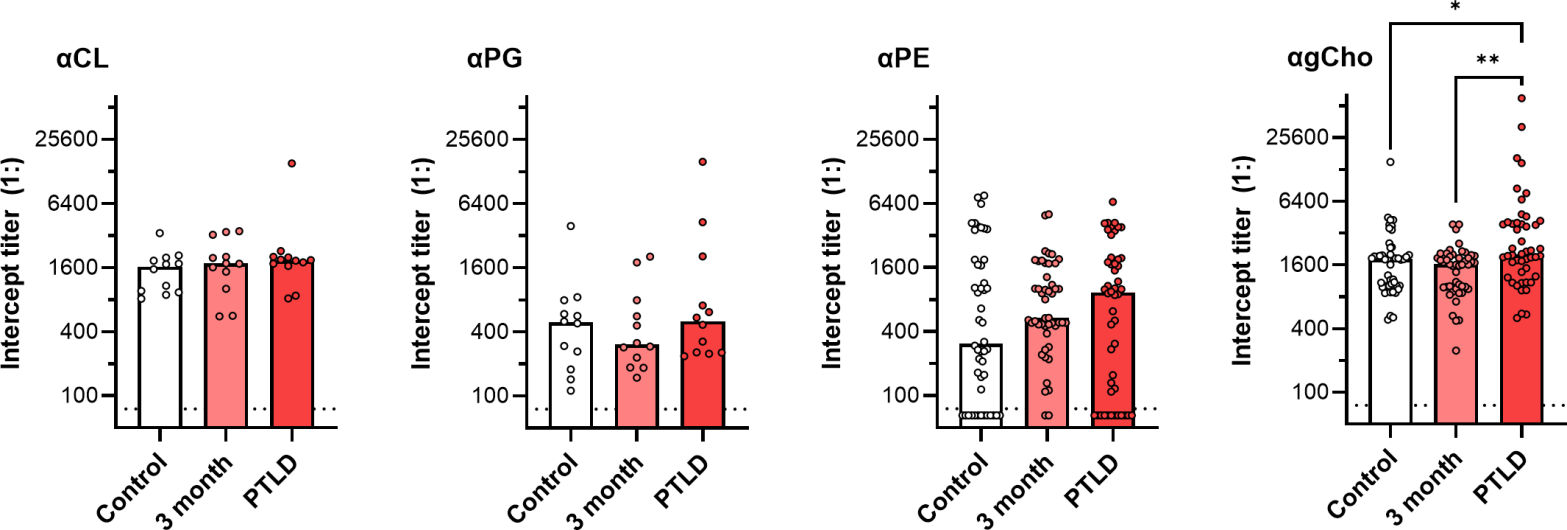
Other lipid antigens in Lyme disease. IgG antibodies to cardiolipin (αCL), phosphatidylglycerol (αPG), phosphatidylethanolamine (αPE), and galactosyl cholesterol (αgCho) were measured. Lyme samples (sourced from Tufts and the LDB) were drawn from patients either 3 months after diagnosis, regardless of symptoms, or >1 year from diagnosis with persistent symptoms. Control samples were endemic controls from the LDB. Groups were either of 12 (αCL, αPG, and αgDAG) or expanded to 48 samples (αPE and αgCho) based on initial differences in the smaller groups. Points are titers from individual patients; bars show the median. *P < 0.05 by Kruskal-Wallis test, **P < 0.01. Comparisons are non-significant (*P* > 0.05) unless indicated. The dashed lines show the lower limits of detection.

We additionally tested for IgG antibodies to three further phospholipids (cardiolipin, phosphatidylglycerol, and phosphatidylethanolamine) and a *B. burgdorferi* glycolipid galactosyl cholesterol (Fig. 2). These experiments were initially performed in groups of 12 patients, selected randomly from the groups described in Fig. 1. These groups were expanded to 48 for two antigens where differences between groups were apparent in preliminary experiments. Titers of αPE increased slightly over time after treatment for Lyme disease, but a large range of values in the controls meant that these increases were not statistically significant. At the later time point, differences in αgCho antibodies were statistically significant by a Kruskal-Wallis test despite a large and overlapping distribution with the controls, likely due to the presence of a long tail of high-titer individuals in the PTLD samples. Galactosyl cholesterol has previously been described as the target of antibodies in Lyme arthritis patients (24). The samples from the Lyme Disease Biobank tested here did not contain any patients with Lyme arthritis, possibly explaining the difference in results between the previous study and this study of PTLD patients.

### Antiphospholipid antibody classes

**Fig 3:**
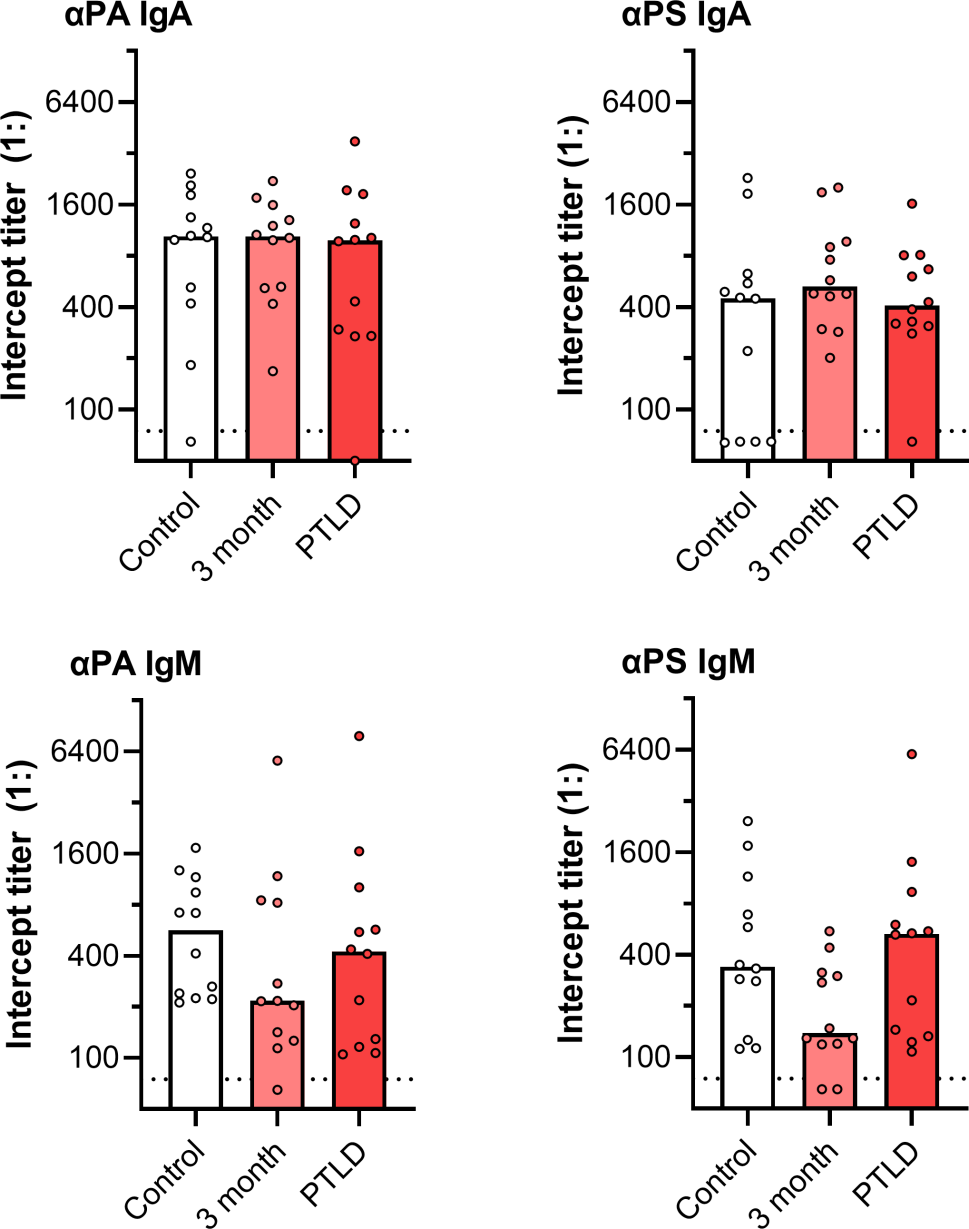
Antiphospholipid IgM and IgA titers in early post-treatment (3 months) and long-term post-treatment Lyme disease (PTLD). IgM and IgA antibodies to phosphatidic acid (αPA) and phosphatidylserine (αPS) were measured in groups of 12 individuals from the Lyme Disease Biobank. Circles are titers from individual patients; bars show the median. All groups were compared to the healthy control using the Kruskal-Wallis test with Dunn’s post hoc, with no significant differences found. Dotted lines represent the limits of detection.

We also determined titers of αPA and αPS IgM and IgA (see Fig. 3). In groups of 12 patients drawn from the same time points as Fig. 1, there were no significant changes in antiphospholipid IgM or IgA. Natural IgM antibodies, including antiphospholipids, contribute to early response to many pathogens, but they serve a coordinating function, and their titers do not typically increase after infection (41). These data agree with previous findings that the IgM response to *B. burgdorferi* is dominated by pathogen-specific circulating IgM, rather than an expansion of B-1-derived natural IgM such as these antiphospholipid antibodies (42). Similarly, while IgA antibodies have been described in Lyme disease (43), there was no change in IgA antiphospholipids. The expansion in antiphospholipid antibodies in Lyme disease is limited to αPA, αPC, and αPS IgG.

### Antiphospholipid IgG over 1 year post-infection

**Fig 4:**
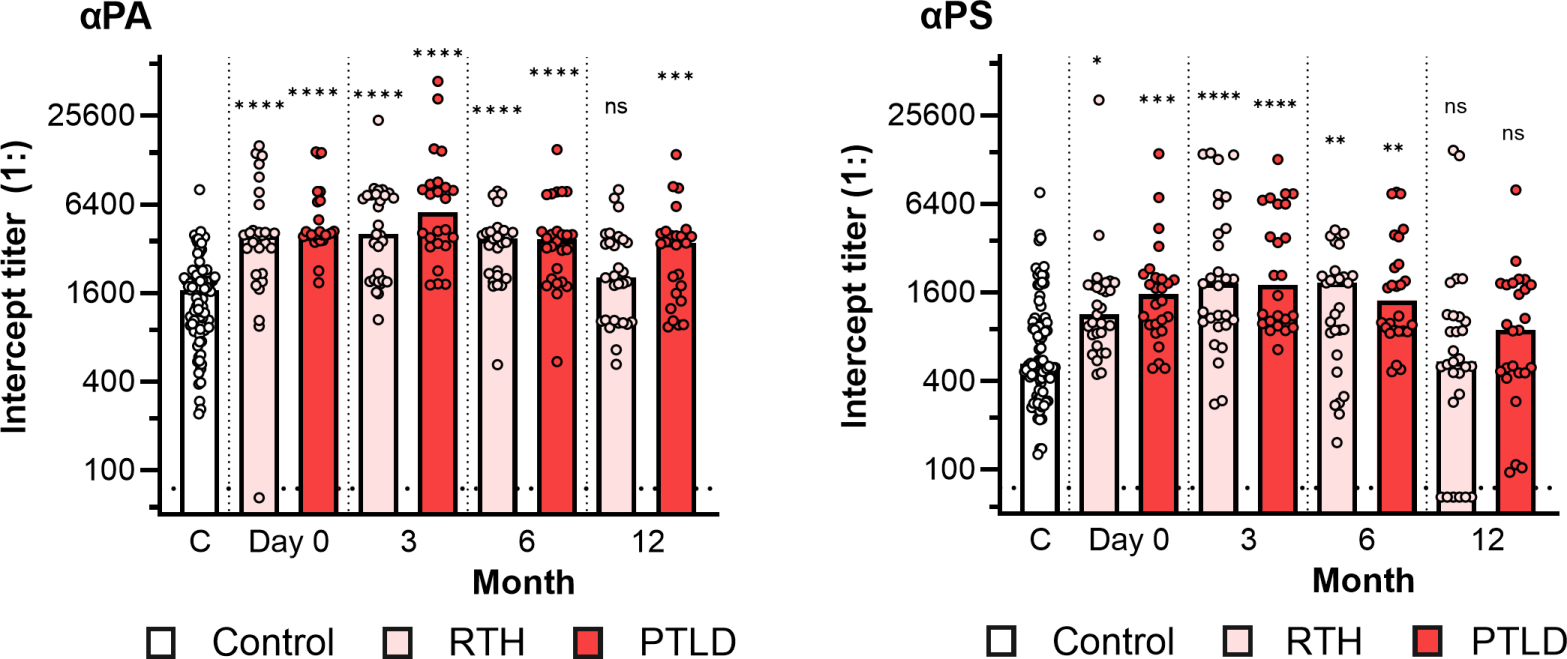
Time course of antiphospholipid antibodies over 1 year of infection. IgG antibodies to phosphatidic acid (αPA) and phosphatidylserine (αPS) were measured over 1 year from diagnosis. Forty-five patients (26 who returned to health after 1 year [RTH] and 29 diagnosed with post-treatment Lyme disease [PTLD] at 1 year) were measured at four time points. Controls were from individuals in endemic areas (48 samples from the LDB) and non-endemic areas (45 samples from Pel-Freez). Points are titers from individual patients; bars show the median. All groups were compared to the healthy control (C) using the Kruskal-Wallis test with Dunn’s post hoc. *P < 0.05, **P < 0.01, ***P << 0.001, ****P < 0.0001. ns, not significantly different from controls. The dashed lines show the lower limits of detection.

Based on the data in Fig. 1–3, αPA and αPS IgG were characterized in serial samples collected over 1 year. Figure 4 compares the kinetics of the antiphospholipid antibody response in two groups: those who recovered at 12 months and those with PTLD symptoms at 6 or 12 months. Regardless of eventual disease outcome, titers are significantly elevated even at day 0, peak at 3–6 months post-infection, and decline toward baseline by 12 months. Although there were small differences between the PTLD and RTH groups at 12 months, the differences between groups at that time point were not significant for either αPA or αPS IgG. This contrasts with the data in Fig. 1, in which long-term sufferers of PTLD (with disease duration of 1–40 years) have elevated αPS. Together, these data suggest that transient induction of antiphospholipid antibodies is a marker of acute infection but that persistent elevation of αPS over longer periods is associated with persistent symptoms.

### Antiphospholipid IgG during acute infection

**Fig 5:**
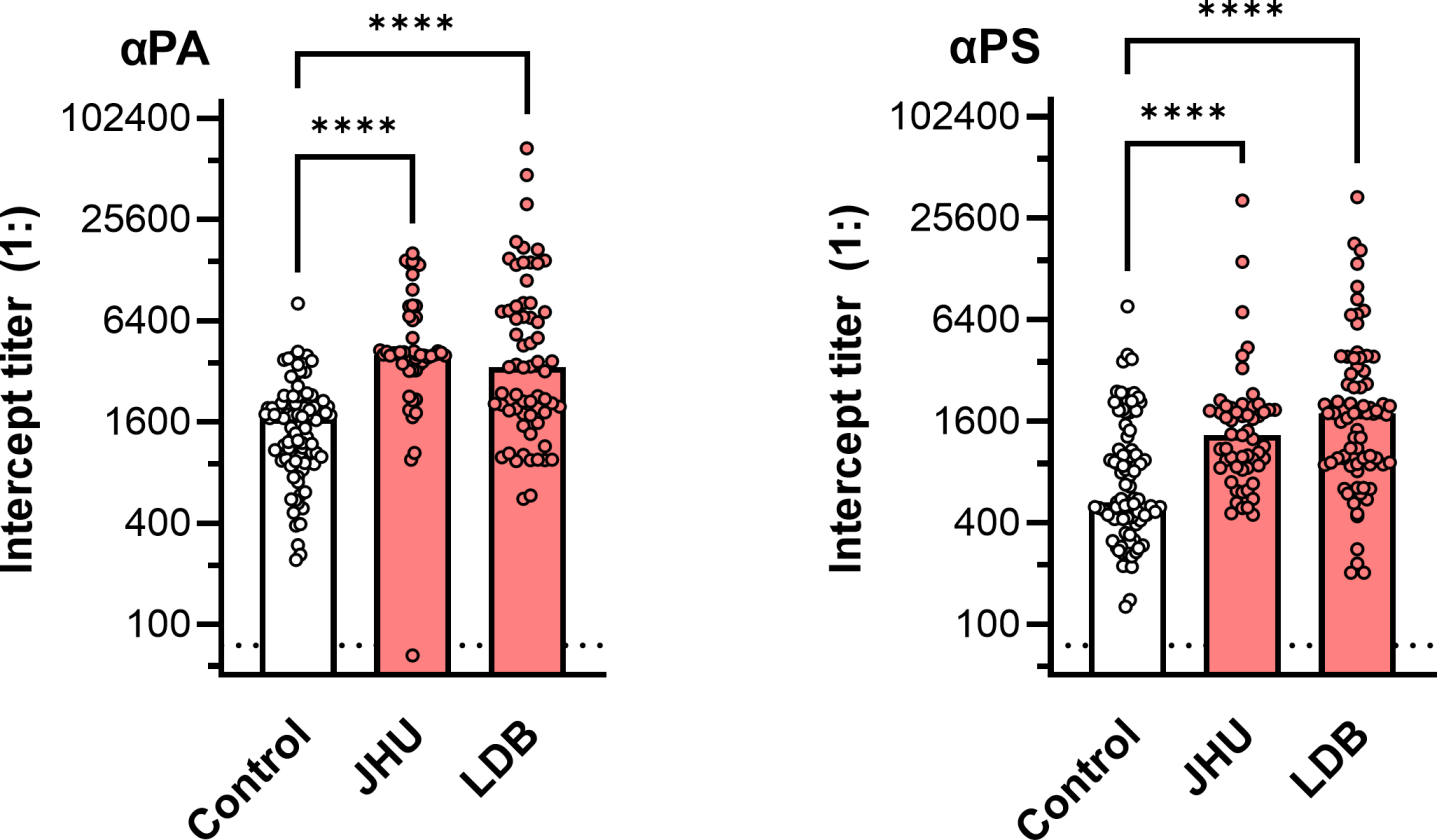
Antiphospholipid titers during acute infection (day 0). IgG antibodies to phosphatidic acid (αPA) and phosphatidylserine (αPS) were measured at study enrollment, within 1 week of initial diagnosis. Samples from two biobanks were compared: 54 from Johns Hopkins University (JHU; the combined day 0 samples from Fig. 3) and 64 from the Lyme Disease Biobank (LDB). Controls were from individuals in endemic areas (48 samples from the LDB) and non-endemic areas (45 samples from Pel-Freez). Points are titers from individual patients; bars show the median. All groups were compared to the healthy control using the Kruskal-Wallis test with Dunn’s post hoc. ****P < 0.0001. The dashed lines show the lower limits of detection.

Most patients analyzed in Fig. 4 had elevated titers of αPA and αPS IgG even at day 0. We further analyzed another 64 day 0 samples from the Lyme Disease Biobank. Antiphospholipid titers in both groups were significantly elevated over controls (Fig. 5), despite the majority of these samples (48 of 64 LDB, 37 of 54 JHU) testing negative by conventional serological diagnostics. Such seronegative acute infections are common in the clinic, and these patients were diagnosed based on clinical presentation and the characteristic erythema migrans rash. These data indicate that antiphospholipid antibodies are detectable earlier than the antibodies used in conventional serology, which are largely raised against borrelial outer membrane proteins. This suggests a potential use for the antiphospholipid antibodies in early diagnosis of infection, where existing diagnostic tests suffer poor sensitivity.

### Antiphospholipid IgG in acute and post-treatment Lyme disease

**Fig 6:**
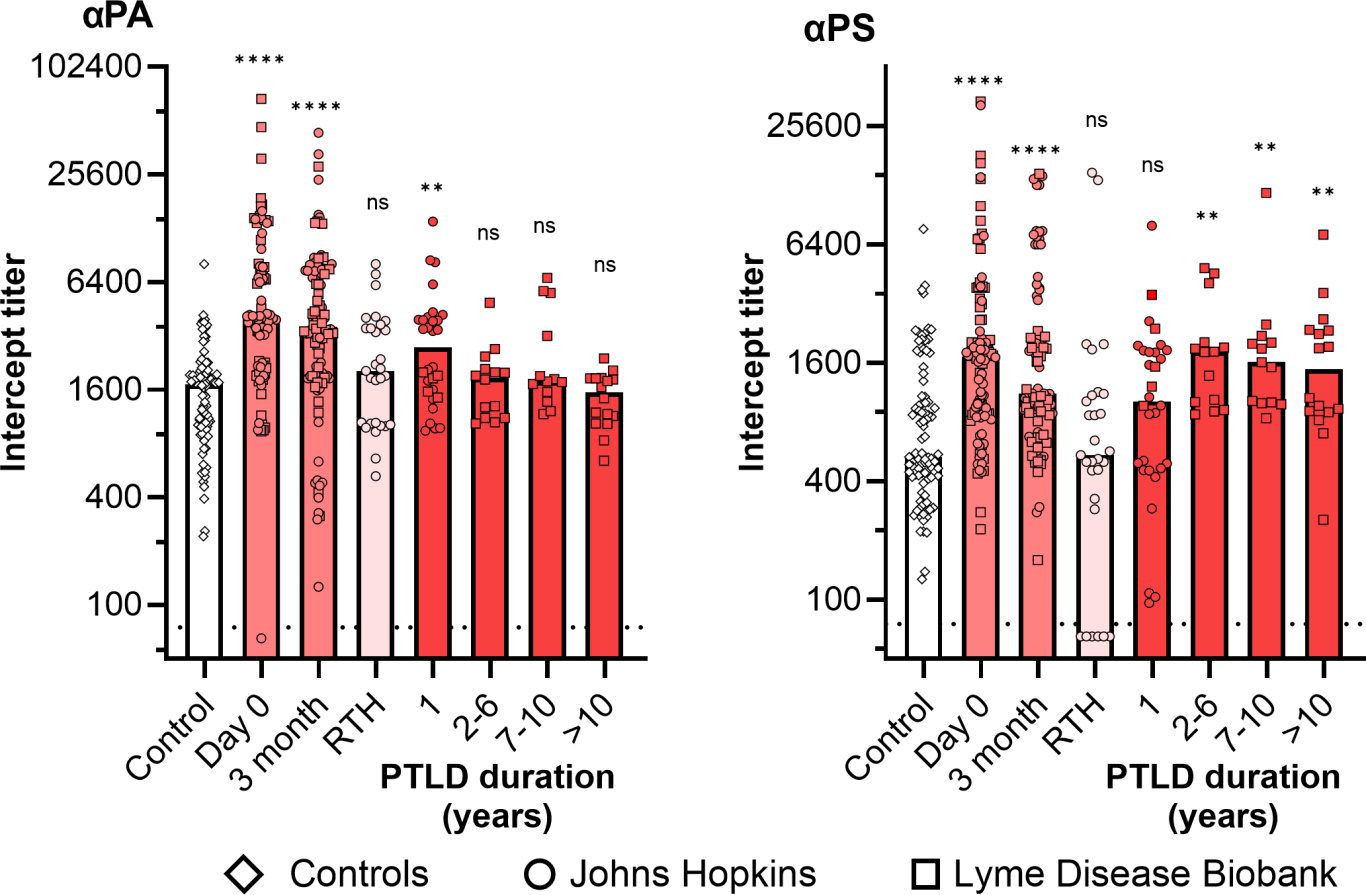
Timeline of antiphospholipid antibodies in acute and post-treatment Lyme disease. IgG antibodies to phosphatidic acid (αPA) and phosphatidylserine (αPS) were measured at several time points during acute infection and after treatment. Samples were drawn from two biobanks: Johns Hopkins University and the Lyme Disease Biobank. Controls were from individuals in endemic areas (48 samples from the LDB) and non-endemic areas (45 samples from Pel-Freez). Circles are titers from individual patients; bars show the median. All groups were compared to the healthy control using the Kruskal-Wallis test with Dunn’s post hoc. **P < 0.01, ****P < 0.0001. The dashed lines show the lower limits of detection.

Figure 6 combines the data presented in Fig. 1, 4, and 5, separating by time since initial diagnosis (at day 0). Figure 6 combines both biobanks: JHU samples are a cohort of 54 individuals drawn serially, while the LDB samples are a mixture of paired (40 samples were paired at day 0 and 3 months) and unique donors. Where similar time points are available (day 0, 3 months, and 1 year post-diagnosis), the titers of samples from both biobanks are similar. Broadly, the titers of αPA IgG are elevated early in disease (day 0 and 3 months post-treatment), before returning to a baseline not statistically different from the controls regardless of outcome (RTH or PTLD). αPS IgG has a similar peak but does not return to baseline in patients with persistent symptoms, even many years after the initial infection. As in all the data sets, there is a wide range of titers within groups, but this likely reflects the heterogeneity of Lyme disease and particularly PTLD patients: each group contains a diversity of symptoms and severities.

### Antiphospholipid IgG in lookalike conditions

**Fig 7:**
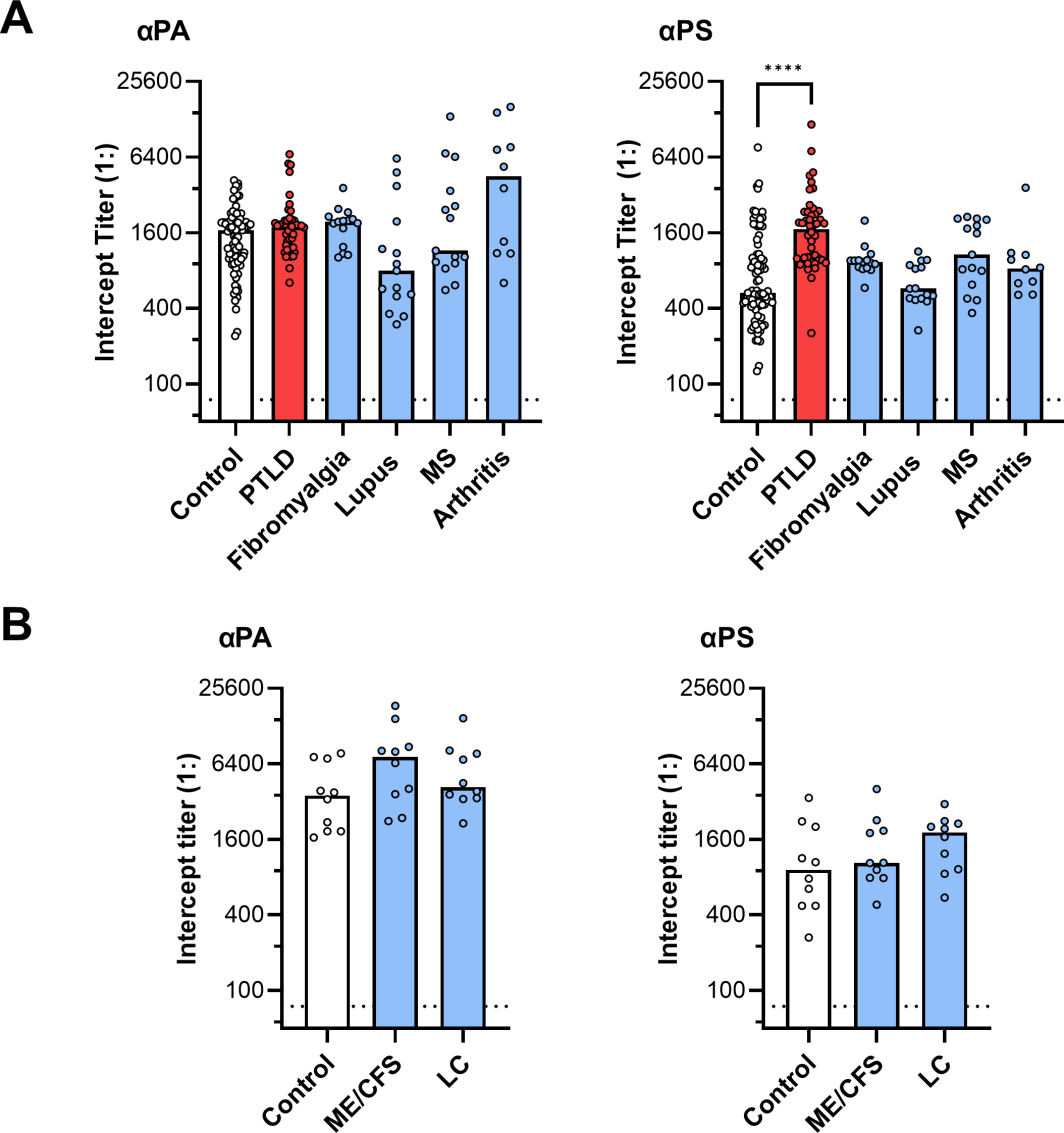
Antiphospholipid antibodies in other autoimmune diseases. IgG antibodies to phosphatidic acid (αPA) and phosphatidylserine (αPS) were measured in serum (**A**) or plasma (**B**) samples from several disease states. Post-treatment Lyme disease (PTLD) was 48 samples from the Lyme Disease Biobank, as presented in Fig. 1. Fibromyalgia, lupus, multiple sclerosis (MS), and rheumatoid arthritis groups were all 15 samples from commercial vendors. Plasma from ME/CFS and long COVID (LC) patients was sourced from the Bateman Horne Center and is compared to control plasma from the same site (*n* = 10 in each group). Circles are titers from individual patients; bars show the median. All groups were compared to the healthy control (C) using the Kruskal-Wallis test with Dunn’s post hoc: differences were non-significant unless denoted. ****P < 0.0001. The dashed lines show the lower limits of detection.

PTLD has symptoms similar to a number of autoimmune conditions, including fibromyalgia, systemic lupus erythematosus, and ME/CFS. Given that phospholipid antibodies have been reported in other autoimmune conditions (16, 17), we assessed the presence of αPA and αPS IgG in some of the above lookalike conditions and others: rheumatoid arthritis, multiple sclerosis, and post-infectious (long) COVID-19 (Fig. 7). The majority of these were measured in serum and compared to the same endemic and non-endemic control sera as in other figures. For chronic fatigue syndrome and post-infectious COVID-19, only plasma samples were available; these were compared to a group of 12 control plasmas. The only group that significantly deviated from the distribution of titers in the control samples was the αPS titers in long-term PTLD patients, as seen in Fig. 1. With the caveat that smaller groups were tested in these other conditions (10 or 15 versus 48 PTLD samples), these data indicate that elevated αPS titers are more common in PTLD than other lookalike conditions. More widespread analysis of autoantibodies in these different conditions is required, but these data suggest that there are different processes driving autoantibody production in Lyme disease than in similar autoimmune conditions.

## DISCUSSION

Previous small studies of Lyme disease have produced scattered descriptions of antilipid antibodies (22, 24, 44, 45), which are known to contribute to pathogenesis in various autoimmune conditions. We conducted a survey of lipid antibodies in acute and post-treatment Lyme disease in order to identify potential drivers of post-infectious chronic symptoms.

Based on our previous studies showing a peak in antibody titer between 1 and 3 months (22), titers of antilipid antibodies were determined in Lyme disease patients at an early post-acute time point (3 months) and in patients >1 year from treatment with PTLD. Only three antibodies were significantly induced after acute infection: PC, the dominant membrane component of *B. burgdorferi*, and two phospholipids (PA and PS) previously shown to be scavenged by the spirochete from its environment (22, 23). PG, which is a minor membrane component and not scavenged, was not antigenic, nor was PE. The origin of the antigens that drive the increased antibody titers is not known. They may be raised after the presentation of bacterial lipids or after inflammation and damage to host tissue. In syphilis, both factors combine to induce anticardiolipin antibodies (46). Examining a time series of αPA and αPS revealed the kinetics of antibody responses over 1 year after diagnosis. IgG αPA and αPS both peak at 3–6 months and decline thereafter.

αPA and αPS were also elevated early in infection in patients with active erythema migrans rashes, before seroconversion on standard tests. Lipid antigens are presented through a different pathway than traditional peptide antigens, using a form of B-cell help driven by iNKT cells (47–49). This pathway, in which innate-like iNKT cells provide cognate help via IL-12 to CD1d-expressing B cells, produces a burst of antibody production without affinity maturation or the production of memory cells. This alternative presentation pathway is consistent with our data in which the majority of patients exhibited a rapid induction of lipid antibodies without a lasting response. Persistent elevation of αPS in some PTLD patients could be due to some dysregulation causing aberrant activation of innate-like B cells or the persistent presence of the PS antigen causing continued activation. While titers of many IgG and IgM antibodies are expected to increase non-specifically during an infection, the response observed here is selective; αPA, αPC, and αPS IgG titers rise without similar elevation in other antilipids.

In PTLD samples collected from patients with a longer interval between diagnosis and collection, a subset of patients identifiable by persistently elevated αPS is apparent. In healthy individuals, antiphospholipid antibodies are typically found as low-affinity “natural” IgM antibodies (50), which serve as an initial innate-like response to pathogen infection (51–53). However, dysregulation of these natural antibodies is common in autoimmune conditions. Although “antiphospholipid” syndrome is diagnosed largely by the presence of antibodies to the lipid-binding protein β_2_GPI (20), actual antiphospholipid antibodies (most commonly αPS and αPE) are also frequently elevated in patients (54). Several infections are known to exacerbate antiphospholipid syndrome, increasing the risk of thromboses and pregnancy loss in sufferers (55). In multiple sclerosis, circulating antilipid antibodies are among the best predictors of severe disease. These include antibodies against both complex glycolipid gangliosides and more common membrane components such as phosphatidylethanolamine and ceramides (56). Similarly, in a survey of systemic lupus erythematosus, levels of serum antilipids (against cardiolipin, cholesterol derivatives, and PS) increased during flares of disease (16). Whether these antibodies, or those identified in this study, directly contribute to pathogenesis or are simply markers of disease is not known; the presence of autoantibodies does not always drive autoimmune disease. More detailed study of the function of antilipid and other autoantibodies in Lyme disease and PTLD could suggest immunomodulatory therapeutic options, such as those that are commonly used in lupus (57).

The clinical relevance of lipid-directed immunity is further underscored by parallels with syphilis, which is caused by the spirochete *Treponema pallidum*. The diagnosis of syphilis is a two-stage algorithm in which antibodies to treponemal surface proteins are used for a highly specific acute-phase diagnostic, followed by less specific antilipid antibodies to monitor progression of disease (58). These adjunct tests (such as the rapid plasma reagin RPR test) detect antibodies against cardiolipin, a phospholipid released from damaged host cells and bacterial membranes during infection (46). In syphilis, these antilipids rise during active infection and decline with successful treatment, providing a dynamic measure of disease activity. The current diagnostic framework for Lyme disease detects acute infections by serology but has no equivalent follow-up test to monitor response to treatment. There may be a role for the use of lipid antibodies as adjuncts to existing diagnostic tests, either to improve early diagnosis or as a measure of response to treatment, as in the non-treponemal tests in syphilis. αPA and αPS were both elevated on the day of enrollment (within 3 days of diagnosis for LDB samples or 1 week for JHU samples) in patients with an erythema migrans, before the development of conventional testing antibodies. Given the ubiquity of phospholipid antibodies in other infections (59–61), the specificity of either antibody may be poor, but existing Lyme disease algorithms already integrate multiple antigens (29). Addition of antilipid antibodies to these panels may improve sensitivity while retaining the more specific diagnostic antibodies. The non-treponemal proof-of-cure tests provide a model for the integration of antilipid assays into clinical care. While the magnitude of the changes in antiphospholipid titers is modest, the degree of elevation is in line with similar quantifications of antiphospholipid antibody titers in other conditions (59, 61, 62). Only a fourfold decrease in titer is considered indicative of cure in syphilis (58).

The availability of clinical samples is a general limitation of many Lyme disease studies. Here, we combine samples from multiple biobanks. The Lyme Disease Biobank is a cross-sectional collection of patients at the point of diagnosis, at follow-up visits around 3 months after diagnosis, and of patients self-reporting persistent symptoms between at least 1 year after diagnosis and treatment. The samples from Johns Hopkins were from a longitudinal study collecting samples at regular intervals over 1 year after diagnosis. Their use in combination allows the timeline of this study to range from the day of diagnosis to the long-term chronic disease that remains a significant health burden (63, 64). Differences in sample collection and storage between these two biobanks and the others used may have accounted for some of the variability seen in antibody titers, but it is notable that where the two biobanks approximately overlap (at day 0, 3 months, and 1 year), their distributions are similar.

This study surveys antilipid antibodies in acute and post-treatment Lyme disease, identifying their transient rise and subsequent decrease in the majority of patients. We identify a subset of patients experiencing chronic symptoms with persistently elevated titers of antiphosphatidylserine. The antibodies described here may be valuable biomarkers of early or persistent disease and suggest another mechanism linking *B. burgdorferi* infection and pathologic autoimmunity.

## SUPPLEMENTAL MATERIAL

Supplemental tables iai.00192-26-s0001.pdf Tables S1 to S5.

ASM does not own the copyrights to Supplemental Material that may be linked to, or accessed through, an article. The authors have granted ASM a non-exclusive, world-wide license to publish the Supplemental Material files. Please contact the corresponding author directly for reuse.
