# Supplementary material for: Antiphospholipid antibodies in acute and post-treatment Lyme disease
Source: Infect Immun. 2026 Jun 30;94(8):e00192-26. doi: 10.1128/iai.00192-26 (PMC13460099; doi:10.1128/iai.00192-26)
Supplement: Supplementary file 1 [file iai.00192-26-s0001.pdf]

		Age			*Sex*		*Race*						*Erythema Migrans (at diagnosis)*		*Lyme Diagnosis*	
**Group**	**Number**	**Range**	**Mean**	**Median**	**Female (%)**	**Male (%)**	**White (%)**	**Black (%)**	**Hispanic (%)**	**East Asian (%)**	**Other or Unknown (%)**	**Collection state**	**Single (%)**	**Multiple (%)**	**STT +ve (%)**	**MTT +ve (%)**
Johns Hopkins Day 0	55	23-83	49	48	60	40	98	0	0	0	2	Maryland	64	36	31	N/A
Johns Hopkins 3 month	55	23-83	49	48	60	40	98	0	0	0	2	Maryland	64	36	44	N/A
Johns Hopkins 6 month	55	23-83	49	48	60	40	98	0	0	0	2	Maryland	64	36	44	N/A
Johns Hopkins 1 year	55	23-83	49	48	60	40	98	0	0	0	2	Maryland	64	36	44	N/A
Lyme Disease Biobank Day 0	64	20-84	53	54	36	64	83	0	17	0	0	New York, Wisconsin	75	12	19	8
Lyme Disease Biobank 3 month	40	20-84	53	54	36	64	83	0	17	0	0	New York, Wisconsin	75	12	20	8
Lyme Disease Biobank PTLD	48	18-81	51	51	83	17	92	0	8	8	0	California, Illinois	15		Historic clinical diagnosis	
Tufts 3 month	8	39-85	53	49	50	50	63	0	0	0	38	Massachusetts	75		25	N/A
Endemic Controls	48	18-78	44	41	65	35	81	2	10	4	2	New York, Wisconsin, Massachusetts	0	0	0	0
Nonendemic Controls	45	18-69	42	39	36	64	0	96	4	0	0	Tennessee	N/A	N/A	N/A	N/A

Supplemental table 1: demographic details of Lyme and control patients. STT = Standard Two-Tiered test, MTT = Modified Two-Tier test. PTLD = Post-treatment Lyme disease. N/A = test not performed

Label	Days since initial visit	Ongoing Lyme Disease Symptoms?	Symptom Survey																										Initially Prescribed Antibiotics for Lyme disease?	Did initial antibiotics resolve symptoms?
			Headache - Current	Headache - Since Last Visit	Fatigue - Current	Fatigue - Since Last Visit	Nightsweats - Current	Nightsweats - Since Last Visit	Flu-like symptoms - Current	Flu-like symptoms - Since Last Visit	Muscle Pain - Current	Muscle Pain - Since Last Visit	Joint Pain - Current	Joint Pain - Since Last Visit	Cardiac/ respiratory - Current	Cardiac/ respiratory - Since Last Visit	GI problems - Current	GI problems - Since Last Visit	Confusion/ memory loss - Current	Confusion/ memory loss - Since Last Visit	Numbness/ tremors - Current	Numbness/ tremors - Since Last Visit	Facial paralysis - Current	Facial paralysis - Since Last Visit	Vision problems - Current	Vision problems - Since Last Visit	Hearing problems - Current	Hearing problems - Since Last Visit		
LDB001	90	Yes	No	No	Yes	Yes	No	No	No	No	No	No	Yes	Yes	No	No	No	No	No	No	No	No	No	No	No	No	No	No	Yes	Yes
LDB002	111	No	No	No	Yes	Yes	No	No	No	No	No	No	No	No	No	No	No	No	No	No	No	No	No	No	No	No	No	No	Yes	Yes
LDB003	143	No	No	No	Yes	Yes	No	No	No	No	No	No	No	Yes	No	No	No	No	No	No	No	No	No	No	No	No	No	No	Yes	Yes
LDB004	185	Yes	Yes	Yes	Yes	Yes	Yes	Yes	Yes	Yes	Yes	Yes	No	No	No	No	No	No	No	No	No	No	No	No	No	No	No	No	Yes	Yes
LDB005	189	Yes	Yes	Yes	Yes	Yes	No	No	No	No	Yes	Yes	No	No	No	No	No	No	No	No	No	No	No	No	No	No	No	No	Yes	Yes
LDB006	93	No	No	No	No	No	No	No	No	No	No	No	No	No	No	No	No	No	No	No	No	No	No	No	No	No	No	No	Yes	Yes
LDB007	71	No	No	No	No	No	No	No	No	No	No	No	No	No	No	No	No	No	No	No	No	No	No	No	No	No	No	No	Yes	Yes
LDB008	87	Yes	No	No	Yes	Yes	No	No	Yes	Yes	Yes	Yes	Yes	Yes	No	No	No	No	No	No	No	No	No	No	No	No	No	No	Yes	No
LDB009	71	No	No	No	No	No	No	No	No	No	No	No	No	No	No	No	No	No	No	No	No	No	No	No	No	No	No	No	No	NA
LDB010	85	No	No	No	No	No	No	No	No	No	No	No	No	No	No	No	No	No	No	No	No	No	No	No	No	No	No	No	Yes	Yes
LDB011	114	Yes	No	No	Yes	Yes	No	No	No	No	No	No	No	No	No	No	Yes	Yes	No	No	No	No	No	No	No	No	No	No	Yes	Yes
LDB012	118	Yes	Yes	No	Yes	No	Yes	No	Yes	No	Yes	No	Yes	No	No	No	No	No	Yes	Yes	No	No	No	No	No	No	No	No	Yes	Yes
LDB013	138	No	No	No	No	No	No	No	No	No	No	No	No	No	No	No	No	No	No	No	No	No	No	No	No	No	No	No	Yes	Yes
LDB014	104	No	No	No	No	No	No	No	No	No	No	No	No	No	No	No	No	No	No	No	No	No	No	No	No	No	No	No	Yes	Yes
LDB015	105	No	No	No	No	No	No	No	No	No	No	No	No	No	No	No	No	No	No	No	No	No	No	No	No	No	No	No	Yes	No
LDB016	91	Yes	No	No	Yes	Yes	No	No	No	No	No	No	No	No	No	No	No	No	No	No	No	No	No	No	No	No	No	No	Yes	Yes
LDB017	116	No	No	No	Yes	Yes	Yes	Yes	No	No	No	No	Yes	Yes	No	No	No	No	No	No	No	No	No	No	No	No	No	No	Yes	Yes
LDB018	65	Yes	No	No	Yes	Yes	No	No	No	No	No	No	Yes	Yes	No	No	No	No	No	No	No	No	No	No	No	No	No	No	Yes	No
LDB019	67	Yes	No	No	No	No	No	No	No	No	Yes	Yes	No	No	No	No	No	No	No	No	No	No	No	No	No	No	No	No	Yes	No
LDB020	87	Yes	No	No	No	No	No	No	No	No	No	No	Yes	Yes	No	No	No	No	No	No	No	No	No	No	No	No	No	No	Yes	Yes
LDB021	82	No	No	No	No	No	No	No	No	No	No	No	No	No	No	No	No	No	No	No	No	No	No	No	No	No	No	No	Yes	Yes
LDB022	91	Yes	Yes	Yes	Yes	Yes	No	No	No	No	Yes	Yes	Yes	Yes	No	No	No	No	No	No	No	No	No	No	No	No	No	No	Yes	Yes
LDB023	147	No	No	No	Yes	Yes	No	No	No	No	No	No	Yes	Yes	No	No	No	No	No	No	No	No	No	No	No	No	No	No	Yes	Yes
LDB024	101	Yes	No	No	No	No	No	No	No	No	No	No	No	No	No	No	No	No	No	No	No	No	No	No	No	No	No	No	Yes	Yes
LDB025	81	Yes	No	No	No	No	No	No	No	No	No	No	No	Yes	No	No	No	No	No	No	No	Yes	No	No	No	No	No	No	Yes	Yes
LDB026	77	No	No	No	Yes	Yes	Yes	Yes	No	No	Yes	Yes	Yes	Yes	No	No	No	No	No	No	No	No	No	No	No	No	No	No	Yes	Yes
LDB027	73	No	No	No	No	No	No	No	No	No	No	No	No	No	No	No	No	No	No	No	No	No	No	No	No	No	No	No	Yes	Yes
LDB028	133	No	No	No	No	No	No	No	No	No	No	No	Yes	Yes	No	No	No	No	No	No	No	No	No	No	No	No	No	No	Yes	Yes
LDB029	141	Yes	No	No	No	No	No	No	No	No	No	No	Yes	Yes	No	No	No	No	No	No	No	No	No	No	No	No	No	No	Yes	Yes
LDB030	86	No	No	No	No	No	No	No	No	No	No	No	No	No	No	No	No	No	No	No	No	No	No	No	No	No	No	No	Yes	Yes
LDB031	83	Yes	Yes	Yes	Yes	Yes	Yes	Yes	No	No	Yes	Yes	No	No	No	No	No	No	No	No	No	No	No	No	No	No	No	No	Yes	Yes
LDB032	104	Yes	No	No	No	No	Yes	No	No	No	No	No	Yes	No	No	No	No	No	No	No	No	No	No	No	No	No	No	No	Yes	Yes
LDB033	104	Yes	No	No	No	No	No	No	No	No	No	No	No	No	No	No	No	No	No	No	No	No	No	No	No	No	No	No	Yes	Yes
LDB034	139	Yes	No	No	No	No	No	No	No	No	Yes	No	Yes	No	No	No	No	No	Yes	No	Yes	No	No	No	No	No	No	No	Yes	Yes
LDB035	84	No	No	No	No	Yes	No	No	No	Yes	No	No	No	No	No	No	No	No	No	No	No	No	No	No	No	No	No	No	Yes	Yes
LDB036	80	Yes	No	No	No	No	No	No	No	No	No	No	Yes	Yes	No	No	No	No	No	No	No	No	No	No	Yes	Yes	No	No	Yes	No
LDB037	71	Yes	No	No	No	No	No	No	No	No	Yes	Yes	Yes	Yes	No	No	No	No	No	No	No	No	No	No	No	No	Yes	Yes	Yes	Yes
LDB038	98	No	No	No	No	No	No	No	No	No	No	No	No	No	No	No	No	No	No	No	No	No	No	No	No	No	No	No	Yes	Yes
LDB039	136	No	No	No	No	No	No	No	No	No	No	No	No	No	No	No	No	No	No	No	No	No	No	No	No	No	No	No	Yes	Yes
LDB040	70	No	No	No	Yes	No	No	No	No	No	No	No	No	No	No	No	No	No	No	No	No	No	No	No	No	No	No	No	Yes	Yes

Table 2: Symptoms reported by 3-month Lyme Disease Biobank patients

Label	Group	I would say that CURRENTLY my general health is...	I would say that AT MY WORST my general health was...	Do you CURRENTLY have the following symptoms related to Lyme Disease																Have you PREVIOUSLY EVER had the following symptoms related to Lyme Disease?															
				Profound Fatigue	Headaches	Joint Pain / Migratory Arthralgias	Musculoskeletal Pain	Neuropathy (numbness/tingling)	Twitching	Memory Loss	Cognitive Impairment	Sleep Impairment	Heart Block / Lyme Carditis	Psychiatric (depression/ mood swings)	Flu-like symptoms (fever/chills/night seats)	Nausea/Gastrointestinal	Sensitivity to Light/Sound/Smells	Vision Problems	Facial Paralysis	Profound Fatigue	Headaches	Joint Pain / Migratory Arthralgias	Musculoskeletal Pain	Neuropathy (numbness/tingling)	Twitching	Memory Loss	Cognitive Impairment	Sleep Impairment	Heart Block / Lyme Carditis	Psychiatric (depression/ mood swings)	Flu-like symptoms (fever/chills/night seats)	Nausea/Gastrointestinal	Sensitivity to Light/Sound/Smells	Vision Problems	Facial Paralysis
LDB102	PTLD	Fair	Poor	No	No	No	Yes	No	No	No	No	No	No	No	No	Yes	No	Yes	No	No	No	Yes	Yes	Yes	Yes	Yes	Yes	No	No	No	No	Yes	No	Yes	No
LDB103	PTLD	Fair	Poor	No	No	Yes	Yes	Yes	No	No	No	Yes	Yes	No	Yes	No	No	No	No	No	Yes	Yes	Yes	Yes	Yes	No	No	Yes	Yes	No	Yes	Yes	No	No	No
LDB112	PTLD	Good	Poor	No	No	No	No	No	No	Yes	No	Yes	No	Yes	No	No	Yes	No	No	Yes	Yes	Yes	Yes	Yes	Yes	Yes	Yes	Yes	Yes	Yes	Yes	Yes	Yes	Yes	Yes
LDB113	PTLD	Fair	Poor	No	No	No	No	No	No	No	Yes	No	No	Yes	No	Yes	No	No	No	Yes	Yes	Yes	No	No	No	No	Yes	Yes	No	Yes	Yes	Yes	No	Yes	x
LDB119	PTLD	x	x	No	No	No	No	Yes	No	No	No	Yes	No	Yes	No	No	No	No	No	Yes	No	Yes	Yes	Yes	No	Yes	Yes	Yes	No	Yes	No	Yes	No	No	No
LDB121	PTLD	x	x	No	Yes	Yes	Yes	Yes	No	Yes	Yes	Yes	No	Yes	Yes	Yes	Yes	No	Yes	Yes	Yes	Yes	Yes	Yes	No	Yes	Yes	Yes	No	Yes	Yes	No	Yes	No	Yes
LDB127	PTLD	x	x	No	Yes	Yes	Yes	Yes	Yes	Yes	Yes	Yes	Yes	No	Yes	Yes	No	Yes	No	Yes	Yes	Yes	Yes	Yes	Yes	Yes	Yes	Yes	Yes	Yes	Yes	Yes	Yes	Yes	No
LDB128	PTLD	x	x	No	Yes	Yes	Yes	No	Yes	No	Yes	Yes	No	Yes	No	Yes	No	No	No	Yes	Yes	Yes	Yes	No	Yes	No	Yes	Yes	No	Yes	Yes	Yes	No	No	No
LDB129	PTLD	Fair	Poor	No	No	No	No	No	Yes	Yes	Yes	Yes	No	Yes	No	No	Yes	Yes	No	No	Yes	No	No	Yes	Yes	Yes	Yes	Yes	No	Yes	Yes	Yes	Yes	Yes	No
LDB131	PTLD	Very Good	Good	No	No	Yes	Yes	No	Yes	No	No	Yes	No	No	No	No	No	No	No	No	No	No	No	No	No	No	No	No	No	No	No	No	No	No	No
LDB133	PTLD	Very Good	Poor	No	No	Yes	No	Yes	Yes	No	No	Yes	No	No	No	Yes	No	No	No	No	No	Yes	No	Yes	Yes	No	Yes	Yes	No	No	Yes	Yes	No	No	No
LDB135	PTLD	Very Good	Fair	No	No	Yes	Yes	Yes	Yes	No	Yes	No	No	Yes	No	Yes	No	No	No	No	No	Yes	Yes	Yes	Yes	No	Yes	No	No	Yes	No	Yes	No	No	No
LDB139	PTLD	Good	Poor	No	Yes	Yes	Yes	No	No	Yes	Yes	Yes	No	No	No	No	Yes	Yes	No	Yes	No	No	No	No	No	No	No	Yes	No	No	Yes	No	Yes	No	No
LDB143	PTLD	Good	Poor	No	Yes	No	Yes	No	No	No	No	No	No	Yes	No	Yes	Yes	No	No	Yes	Yes	No	Yes	Yes	Yes	Yes	Yes	Yes	No	Yes	Yes	Yes	Yes	No	No
LDB145	PTLD	Very Good	Poor	No	No	Yes	No	Yes	No	Yes	No	Yes	No	Yes	No	No	Yes	Yes	No	Yes	Yes	Yes	Yes	Yes	Yes	Yes	Yes	Yes	Yes	Yes	Yes	Yes	Yes	Yes	No
LDB101	PTLD	Fair	Poor	Yes	Yes	No	Yes	No	No	Yes	Yes	Yes	No	Yes	Yes	No	No	Yes	No	Yes	Yes	No	Yes	No	No	Yes	Yes	Yes	No	Yes	Yes	No	No	Yes	No
LDB104	PTLD	Poor	Poor	Yes	Yes	Yes	Yes	Yes	No	Yes	Yes	Yes	No	Yes	Yes	Yes	Yes	Yes	No	Yes	Yes	Yes	Yes	Yes	No	Yes	Yes	Yes	No	Yes	Yes	Yes	Yes	Yes	No
LDB105	PTLD	Good	Fair	Yes	No	Yes	Yes	No	No	Yes	No	No	No	No	No	No	Yes	No	No	Yes	No	No	Yes	No	Yes	Yes	Yes	Yes	No	No	Yes	No	Yes	Yes	No
LDB106	PTLD	Fair	Poor	Yes	Yes	Yes	No	No	No	Yes	Yes	Yes	Yes	No	No	Yes	Yes	No	No	Yes	Yes	Yes	Yes	Yes	Yes	Yes	Yes	Yes	Yes	Yes	Yes	Yes	Yes	Yes	No
LDB107	PTLD	Fair	Poor	Yes	Yes	Yes	Yes	Yes	No	Yes	Yes	Yes	No	Yes	No	No	Yes	Yes	No	Yes	Yes	Yes	Yes	Yes	Yes	Yes	Yes	Yes	No	Yes	No	No	Yes	Yes	No
LDB108	PTLD	Good	Poor	Yes	No	Yes	Yes	Yes	Yes	Yes	Yes	Yes	No	No	Yes	Yes	Yes	Yes	No	Yes	No	Yes	Yes	Yes	Yes	Yes	Yes	Yes	No	Yes	Yes	Yes	Yes	Yes	No
LDB109	PTLD	Fair	Poor	Yes	Yes	Yes	Yes	Yes	Yes	Yes	Yes	No	No	No	Yes	Yes	Yes	Yes	No	Yes	Yes	Yes	Yes	Yes	Yes	Yes	Yes	No	No	Yes	Yes	Yes	Yes	Yes	Yes
LDB110	PTLD	Fair	Poor	Yes	Yes	Yes	No	Yes	No	Yes	Yes	Yes	No	No	No	No	Yes	No	No	Yes	Yes	Yes	Yes	Yes	Yes	Yes	Yes	Yes	No	No	Yes	Yes	Yes	No	No
LDB111	PTLD	Good	Poor	Yes	Yes	Yes	Yes	Yes	No	No	Yes	Yes	No	Yes	Yes	Yes	No	Yes	No	Yes	Yes	Yes	Yes	Yes	No	Yes	Yes	Yes	No	Yes	Yes	Yes	No	Yes	No
LDB114	PTLD	Poor	Poor	Yes	Yes	Yes	Yes	Yes	Yes	Yes	Yes	Yes	No	Yes	No	Yes	Yes	Yes	No	Yes	Yes	Yes	Yes	Yes	No	Yes	Yes	Yes	No	Yes	No	Yes	Yes	Yes	No
LDB115	PTLD	Poor	Poor	Yes	Yes	Yes	Yes	Yes	Yes	Yes	Yes	Yes	Yes	No	Yes	Yes	Yes	Yes	No	Yes	Yes	Yes	Yes	Yes	Yes	Yes	Yes	Yes	Yes	Yes	Yes	Yes	Yes	Yes	No
LDB116	PTLD	x	x	Yes	No	Yes	Yes	Yes	Yes	Yes	Yes	Yes	No	Yes	Yes	Yes	Yes	No	No	Yes	Yes	Yes	Yes	Yes	Yes	Yes	Yes	Yes	No	Yes	Yes	Yes	Yes	Yes	Yes
LDB117	PTLD	x	x	Yes	Yes	Yes	No	Yes	No	No	No	No	No	Yes	Yes	Yes	Yes	No	No	Yes	Yes	Yes	Yes	Yes	No	Yes	Yes	Yes	No	Yes	Yes	Yes	Yes	Yes	No
LDB118	PTLD	Poor	Poor	Yes	No	Yes	Yes	No	Yes	Yes	Yes	No	No	No	Yes	Yes	Yes	Yes	No	Yes	No	Yes	Yes	No	Yes	Yes	Yes	Yes	No	No	Yes	No	No	No	No
LDB120	PTLD	x	x	Yes	No	Yes	Yes	Yes	No	Yes	Yes	Yes	No	Yes	No	Yes	Yes	Yes	No	Yes	Yes	Yes	Yes	Yes	No	Yes	Yes	Yes	No	Yes	No	Yes	Yes	Yes	No
LDB122	PTLD	x	x	Yes	Yes	Yes	Yes	Yes	No	Yes	Yes	Yes	No	Yes	Yes	Yes	Yes	No	No	Yes	Yes	Yes	Yes	No	No	Yes	Yes	Yes	No	Yes	Yes	No	Yes	No	No
LDB123	PTLD	x	x	Yes	No	Yes	Yes	Yes	Yes	Yes	Yes	Yes	No	Yes	Yes	Yes	Yes	Yes	Yes	Yes	Yes	Yes	Yes	No	No	Yes	Yes	Yes	No	Yes	Yes	Yes	No	No	No
LDB124	PTLD	x	x	Yes	Yes	Yes	Yes	Yes	Yes	No	No	Yes	No	No	Yes	Yes	Yes	No	No	Yes	Yes	Yes	Yes	Yes	Yes	Yes	Yes	Yes	No	No	Yes	Yes	Yes	Yes	Yes
LDB125	PTLD	x	x	Yes	Yes	Yes	Yes	Yes	No	No	Yes	Yes	No	No	Yes	No	Yes	No	No	Yes	Yes	Yes	Yes	Yes	Yes	Yes	Yes	Yes	Yes	Yes	Yes	Yes	Yes	No	No
LDB126	PTLD	x	x	Yes	Yes	Yes	Yes	Yes	No	Yes	Yes	Yes	No	Yes	Yes	No	Yes	No	x	Yes	Yes	Yes	Yes	Yes	No	Yes	Yes	Yes	No	Yes	Yes	Yes	Yes	No	No
LDB130	PTLD	Fair	Poor	Yes	Yes	No	No	Yes	Yes	No	Yes	Yes	No	Yes	Yes	No	Yes	Yes	No	Yes	Yes	No	No	No	No	No	Yes	Yes	No	Yes	Yes	No	No	Yes	No
LDB132	PTLD	Fair	Poor	Yes	Yes	Yes	Yes	Yes	Yes	Yes	Yes	Yes	No	Yes	Yes	Yes	Yes	No	No	Yes	No	Yes	Yes	Yes	Yes	Yes	Yes	Yes	No	Yes	Yes	Yes	Yes	No	No
LDB134	PTLD	Good	Poor	Yes	Yes	Yes	Yes	No	Yes	Yes	Yes	Yes	No	Yes	No	Yes	Yes	No	Yes	Yes	No	Yes	Yes	No	Yes	Yes	Yes	Yes	No	Yes	Yes	No	Yes	No	Yes
LDB136	PTLD	Fair	Poor	Yes	Yes	No	Yes	Yes	Yes	Yes	Yes	Yes	No	Yes	No	Yes	Yes	Yes	No	Yes	Yes	Yes	Yes	Yes	Yes	Yes	Yes	Yes	No	Yes	Yes	Yes	Yes	Yes	No
LDB137	PTLD	Fair	Poor	Yes	Yes	Yes	Yes	No	Yes	Yes	No	Yes	No	Yes	No	Yes	Yes	Yes	No	Yes	Yes	Yes	Yes	No	Yes	No	No	No	No	Yes	No	No	Yes	No	No
LDB138	PTLD	Fair	Fair	Yes	No	Yes	Yes	No	No	Yes	Yes	Yes	No	No	Yes	Yes	No	No	No	Yes	Yes	Yes	Yes	No	No	Yes	Yes	Yes	No	No	Yes	Yes	No	No	No
LDB140	PTLD	Good	Poor	Yes	Yes	Yes	No	No	No	Yes	Yes	No	No	Yes	Yes	Yes	Yes	Yes	No	No	No	Yes	Yes	No	No	No	No	No	No	No	Yes	Yes	Yes	Yes	No
LDB141	PTLD	Good	Poor	Yes	No	Yes	No	Yes	No	Yes	No	Yes	No	No	No	No	No	Yes	No	Yes	Yes	Yes	Yes	Yes	Yes	Yes	Yes	Yes	Yes	Yes	Yes	Yes	Yes	Yes	Yes
LDB142	PTLD	Poor	Poor	Yes	Yes	No	Yes	No	Yes	No	No	Yes	Yes	No	Yes	Yes	Yes	No	No	Yes	Yes	No	Yes	No	Yes	No	No	Yes	Yes	No	No	Yes	No	No	No
LDB144	PTLD	Fair	Poor	Yes	No	Yes	Yes	No	Yes	Yes	Yes	Yes	No	No	No	Yes	No	No	No	Yes	Yes	Yes	Yes	No	Yes	Yes	Yes	Yes	No	Yes	No	Yes	No	No	No
LDB146	PTLD	Very Good	Poor	Yes	No	Yes	Yes	No	No	Yes	Yes	Yes	No	No	No	Yes	No	No	No	Yes	Yes	Yes	Yes	No	No	Yes	Yes	Yes	No	No	Yes	Yes	No	No	No
LDB147	PTLD	Poor	Poor	Yes	Yes	Yes	Yes	Yes	Yes	Yes	Yes	No	No	Yes	No	Yes	Yes	Yes	Yes	Yes	Yes	Yes	Yes	Yes	Yes	Yes	Yes	No	Yes	Yes	Yes	Yes	Yes	Yes	Yes
LDB148	PTLD	Good	Poor	Yes	Yes	No	Yes	Yes	No	No	Yes	Yes	No	No	Yes	Yes	No	No	No	Yes	Yes	No	Yes	No	No	Yes	Yes	Yes	No	Yes	Yes	Yes	Yes	Yes	No

Table 3: Symptoms reported by Lyme Disease Biobank PTLD patients

Label	Group	Time point	Fatigue severity scale total score	McGill Pain Questionnaire: McGill total score	McGill affective score	McGill sensory score	Pittsburgh sleep quality index	Beck Depression Index: Beck total score	Beck affective score	Beck sensory score	SF-36 Health Survey: Physical component score	Mental component score	Mental health score	Role-emotional (RE) score	Societal function score	Vitality score	General health score	Bodily pain score	Role-physical (RP) score	Physical function score
JHU001	RTH	6 month	15	5	2	3	5	5	4	1	54.61	48.4	52.82	51.99	45.94	48.97	50.55	50.29	56.85	57.03
JHU002	PTLD	6 month	9	0	0	0	2	0	0	0	57.34	57.29	61.27	55.88	56.85	52.09	63.9	62.12	54.4	54.93
JHU003	PTLD	6 month	34	5	2	3	8	5	2	3	37.57	61.31	55.64	55.88	56.85	45.85	57.7	37.61	47.06	33.88
JHU004	RTH	6 month	16	2	0	2	3	0	0	0	50.93	56.61	52.82	55.88	56.85	58.33	50.55	46.06	56.85	52.82
JHU005	PTLD	6 month	41	2	1	1	8	10	7	3	54.96	30.05	38.74	28.67	35.03	39.6	57.7	55.36	37.26	48.61
JHU006	PTLD	6 month	40	0	0	0	11	16	11	5	58.78	40.61	41.56	55.88	40.49	42.72	45.78	62.12	56.85	57.03
JHU007	RTH	6 month	18	0	0	0	3	0	0	0	56.14	54.2	55.64	55.88	56.85	48.97	62.47	50.29	56.85	57.03
JHU008	RTH	6 month	27	0	0	0	6	10	10	0	59.59	53.96	52.82	55.88	56.85	58.33	57.7	62.12	56.85	57.03
JHU009	RTH	6 month	18	0	0	0	0	0	0	0	58.35	56.7	58.46	55.88	56.85	58.33	57.7	62.12	56.85	57.03
JHU010	PTLD	6 month	41	16	1	15	10	7	3	4	41.43	50.82	55.64	55.88	40.49	33.36	52.93	37.18	42.16	50.72
JHU011	RTH	6 month	23	0	0	0	5	0	0	0	60.4	49.89	52.82	48.1	56.85	55.21	55.32	62.12	56.85	57.03
JHU012	RTH	6 month	25	3	1	2	6	6	4	2	47.37	47.45	47.19	55.88	40.49	45.85	45.78	46.06	42.16	57.03
JHU013	RTH	6 month	15	2	0	2	8	4	1	3	59.85	45.56	41.56	48.1	56.85	55.21	55.32	55.36	56.85	54.93
JHU014	RTH	6 month	22	1	0	1	6	15	11	4	47.42	44.71	41.56	51.99	40.49	48.97	38.63	51.13	47.06	48.61
JHU015	RTH	6 month	16	0	0	0	7	1	0	1	62.01	46.21	52.82	44.22	45.94	58.33	61.51	62.12	54.4	57.03
JHU016	RTH	6 month	35	0	0	0	.	0	0	0	61.14	53.87	52.82	55.88	56.85	58.33	63.9	62.12	56.85	57.03
JHU017	RTH	6 month	10	0	0	0	1	0	0	0	59.01	62.28	64.09	55.88	56.85	70.82	63.9	62.12	56.85	57.03
JHU018	RTH	6 month	18	0	0	0	4	1	1	0	59.29	51.85	47.19	55.88	56.85	58.33	60.08	55.36	56.85	57.03
JHU019	PTLD	6 month	36	4	0	4	7	15	10	5	44.93	38.01	50.01	28.67	29.58	45.85	50.55	50.29	29.91	44.41
JHU020	RTH	6 month	14	0	0	0	4	0	0	0	56.56	57.33	58.46	55.88	56.85	58.33	59.13	55.36	56.85	57.03
JHU021	PTLD	6 month	55	13	2	11	15	.	28	.	48.71	18.35	19.03	28.67	24.13	30.24	42.45	51.13	29.91	42.3
JHU022	PTLD	6 month	37	8	2	6	6	10	7	3	53.43	35.25	44.38	32.56	40.49	42.72	50.55	37.61	51.96	54.93
JHU023	RTH	6 month	21	2	0	2	3	0	0	0	52.69	56.86	58.46	55.88	56.85	58.33	54.36	55.36	56.85	50.72
JHU024	PTLD	6 month	18	2	0	2	1	0	0	0	55.08	58.19	58.46	55.88	56.85	58.33	55.32	62.12	56.85	50.72
JHU025	PTLD	6 month	28	12	1	11	8	20	14	6	50.59	39.81	44.38	44.22	40.49	39.6	50.55	41.41	47.06	54.93
JHU026	RTH	6 month	16	0	0	0	2	0	0	0	59.55	60.17	61.27	55.88	56.85	67.7	63.9	62.12	56.85	57.03
JHU027	RTH	6 month	9	0	0	0	3	1	0	1	57.32	59.57	61.27	55.88	56.85	64.58	55.32	62.12	56.85	57.03
JHU028	PTLD	6 month	31	7	3	4	6	11	6	5	41.34	53.77	47.19	51.99	51.4	52.09	43.4	46.06	44.61	40.2
JHU029	PTLD	6 month	.	2	0	2	4	11	8	3	56.6	25.07	27.48	32.56	35.03	39.6	50.55	41.41	47.06	54.93
JHU030	PTLD	6 month	44	6	4	2	8	15	11	4	47.55	36.36	38.74	44.22	40.49	33.36	41.02	41.41	42.16	54.93
JHU031	RTH	6 month	15	0	0	0	11	3	0	3	56.74	57.5	55.64	55.88	56.85	61.46	59.13	62.12	56.85	50.72
JHU032	PTLD	6 month	46	11	1	10	6	14	9	5	44.16	44.13	47.19	48.1	35.03	42.72	50.55	41.41	34.81	52.82
JHU033	PTLD	6 month	36	6	1	5	4	14	9	5	47.27	44.39	47.19	48.1	45.94	33.36	55.32	46.06	42.16	48.61
JHU034	RTH	6 month	14	0	0	0	2	4	3	1	56.21	50.74	52.82	51.99	56.85	48.97	45.78	62.12	56.85	54.93
JHU035	RTH	6 month	21	1	0	1	7	1	1	0	57.25	57.51	58.46	55.88	56.85	61.46	52.93	62.12	56.85	57.03
JHU036	PTLD	6 month	47	9	2	7	9	14	9	5	48.33	35.69	44.38	36.44	40.49	30.24	48.17	46.06	39.71	50.72
JHU037	PTLD	6 month	19	6	2	4	8	26	20	6	60.22	10.95	13.4	20.89	35.03	27.11	52.93	46.06	37.26	54.93
JHU038	PTLD	6 month	58	10	3	7	14	16	10	6	40.63	34.67	35.93	48.1	29.58	27.11	30.53	37.18	47.06	44.41
JHU039	RTH	6 month	9	0	0	0	3	0	0	0	57.9	54.81	52.82	55.88	56.85	61.46	50.55	62.12	56.85	57.03
JHU040	PTLD	6 month	29	2	1	1	3	5	2	3	44.63	46.57	44.38	44.22	45.94	48.97	48.17	46.06	42.16	42.3
JHU041	RTH	6 month	24	1	0	1	2	1	1	0	57.34	58.24	58.46	55.88	56.85	64.58	52.93	62.12	56.85	57.03
JHU042	PTLD	6 month	41	14	3	11	5	23	14	9	48.55	25.53	30.3	28.67	40.49	23.99	45.78	37.61	42.16	46.51
JHU043	PTLD	6 month	28	5	0	5	3	20	16	4	52.58	29.85	41.56	24.78	35.03	39.6	50.55	50.29	37.26	50.72
JHU044	PTLD	6 month	23	6	0	6	8	6	1	5	43.77	38.98	44.38	40.33	35.03	39.6	41.02	41.41	37.26	50.72
JHU045	PTLD	6 month	52	2	0	2	10	13	6	7	41.3	44.37	47.19	48.1	40.49	36.48	36.25	41.83	42.16	48.61
JHU046	RTH	6 month	30	5	0	5	3	2	0	2	43.49	57.04	52.82	55.88	51.4	55.21	52.93	37.18	42.16	52.82
JHU047	RTH	6 month	19	0	0	0	8	7	6	1	64.59	35.81	41.56	32.56	45.94	58.33	52.93	62.12	54.4	57.03
JHU048	RTH	6 month	.	0	0	0	1	0	0	0	56.98	44.52	52.82	36.44	45.94	58.33	52.93	51.13	54.4	54.93
JHU049	PTLD	6 month	42	2	0	2	.	0	0	0	48.56	49.83	55.64	48.1	45.94	45.85	50.55	50.29	44.61	52.82
JHU050	RTH	6 month	18	0	0	0	3	4	1	3	49.02	51.74	52.82	48.1	56.85	48.97	43.4	55.36	44.61	52.82
JHU051	PTLD	6 month	50	3	0	3	5	11	3	8	34.02	53.52	52.82	55.88	56.85	42.72	45.78	41.41	32.36	40.2
JHU052	RTH	6 month	22	0	0	0	4	0	0	0	50.77	63.86	64.09	55.88	56.85	67.7	50.55	55.36	56.85	50.72
JHU053	PTLD	6 month	40	6	1	5	5	9	4	5	26.2	54.92	52.82	44.22	45.94	39.6	37.68	41.83	29.91	23.36
JHU054	RTH	6 month	9	0	0	0	1	4	2	2	52.61	60.62	61.27	55.88	56.85	58.33	60.08	55.36	56.85	57.03
JHU055	RTH	6 month	13	2	0	2	4	0	0	0	54.62	56.52	55.64	55.88	56.85	58.33	55.32	50.29	56.85	57.03

Label	Post-Lyme Questionnaire of Symptoms (0=absent, 1=mild, 2=moderate, 3=severe): Total score	Fever	Chills	Sweats	Fatigue	Muscle pain	Joint pain	Joint swelling	Numbness in hands/feet	Numbness in face and scalp	Muscle twitching	Headache	Eyes sensitive to light	Vision clarity	Double vision	Dropping face	Drooping eyelid	Dizziness	Ringing in ears	Neck pain	Lower back pain	Poor coordination	Memory impairment	Difficulty winding words	Difficulty focusing or concentrating	Heart palpitations	Difficulty breathing	Swollen lymph nodes	Sore throat	Urination changes	Nausea	Vomiting	Diarrhea	Sleep disrution	Anxiety	Depression	Irritability
JHU001	4	0	0	2	1	1	1	0	2	1	0	2	0	1	0	0	0	0	1	1	0	1	1	1	1	0	0	0	0	0	0	2	1	1	0	0	1
JHU002	0	0	0	0	0	0	0	0	0	0	0	0	0	0	0	0	0	0	0	0	0	0	0	0	0	0	0	0	0	0	0	0	0	0	0	0	0
JHU003	4	1	0	0	2	1	3	2	0	0	1	0	0	0	0	0	0	0	0	0	2	0	0	0	0	0	0	0	0	0	0	0	0	1	1	0	1
JHU004	0	0	0	0	1	1	1	1	0	0	0	1	1	0	0	0	0	0	0	1	1	0	0	1	0	0	0	0	0	0	0	0	1	1	0	0	0
JHU005	1	0	0	0	2	0	1	0	0	0	1	0	0	0	0	0	0	0	0	0	0	0	0	0	0	0	0	0	0	0	0	0	0	1	1	0	0
JHU006	9	2	0	0	2	1	1	0	0	0	1	1	0	1	0	0	0	0	0	0	0	2	2	2	2	0	0	0	3	0	0	0	0	2	1	2	1
JHU007	0	0	0	0	1	1	0	0	0	0	0	0	0	0	0	0	0	0	0	0	0	0	0	0	0	0	0	0	0	0	0	0	0	0	0	0	0
JHU008	0	0	0	0	0	1	0	0	0	0	0	0	0	0	0	0	0	0	0	0	0	0	0	0	0	0	0	0	1	0	0	0	0	0	0	0	0
JHU009	0	0	0	0	0	0	0	0	0	0	0	1	0	0	0	0	0	0	0	0	0	0	0	0	0	0	0	0	1	0	0	0	0	0	0	0	0
JHU010	5	0	0	0	1	3	2	1	2	0	0	1	0	0	0	0	0	0	0	1	3	1	1	2	1	0	0	0	0	0	0	0	0	1	0	0	0
JHU011	0	0	0	0	0	0	0	0	0	0	0	1	0	0	0	0	0	0	0	0	0	0	0	0	0	0	1	0	0	0	0	0	1	1	1	0	0
JHU012	1	2	1	1	1	1	1	1	0	0	0	1	0	0	0	0	0	1	0	1	0	0	0	0	0	1	0	1	1	0	0	0	0	1	1	1	1
JHU013	1	0	0	0	1	0	1	0	0	0	0	0	0	0	0	0	0	0	0	0	0	0	0	1	1	1	0	0	0	0	0	0	1	1	2	1	0
JHU014	1	1	1	1	1	1	1	0	0	0	1	0	1	0	0	0	0	0	2	0	1	1	0	0	0	0	1	1	1	0	0	0	0	1	1	1	0
JHU015	0	0	0	0	1	0	0	0	0	0	0	0	0	0	0	0	0	0	0	0	0	0	0	0	0	0	0	0	0	0	0	0	0	1	0	0	0
JHU016	0	0	0	0	0	0	0	0	0	0	0	0	0	0	0	0	0	0	0	0	0	0	0	0	0	0	0	0	0	0	0	0	0	0	0	0	0
JHU017	0	0	0	0	0	0	0	0	0	0	0	1	0	0	0	0	0	0	0	0	1	0	0	0	0	0	0	0	0	0	0	0	0	0	0	0	0
JHU018	0	0	0	0	1	1	1	0	0	0	0	1	0	0	0	0	0	0	0	1	1	0	0	0	0	0	0	0	0	0	0	0	1	0	0	0	1
JHU019	1	0	0	0	1	0	1	2	0	0	0	0	0	0	0	0	0	1	0	1	1	0	1	1	1	0	0	0	0	0	0	0	0	0	1	1	0
JHU020	0	0	0	0	0	0	0	0	0	0	0	0	0	0	0	0	0	0	0	0	0	0	0	0	0	0	0	0	0	0	0	0	0	0	0	0	0
JHU021	12	0	1	0	2	2	2	0	2	0	2	1	1	0	0	0	0	1	1	2	2	1	1	1	1	1	2	0	1	0	1	1	1	3	2	2	2
JHU022	4	0	0	1	2	2	1	0	0	0	0	1	1	0	0	0	0	0	0	2	2	0	0	0	1	0	0	0	0	0	0	0	0	0	1	1	1
JHU023	0	0	0	1	0	1	1	0	1	0	0	0	0	0	0	0	0	0	0	0	1	0	0	1	1	0	0	0	0	0	0	0	0	0	0	1	0
JHU024	0	0	0	0	1	1	1	1	0	1	0	1	1	1	0	0	0	0	0	0	0	0	0	0	0	0	0	0	0	0	0	0	0	0	0	0	0
JHU025	13	0	0	2	2	2	3	1	0	0	0	2	2	0	0	0	0	0	0	0	2	1	2	2	2	0	2	2	1	0	1	0	0	0	2	1	0
JHU026	1	0	0	0	0	0	0	1	0	0	0	0	0	0	0	0	0	0	2	0	0	0	0	0	0	0	0	0	0	0	0	0	0	0	1	0	0
JHU027	0	0	0	0	1	1	0	0	0	0	1	1	0	0	0	0	0	0	0	0	1	0	0	0	0	0	0	0	0	0	0	0	0	0	0	0	0
JHU028	7	0	0	0	2	0	2	1	0	0	0	0	1	0	0	0	0	0	0	2	2	0	1	2	1	2	0	0	1	0	0	0	2	0	1	1	0
JHU029	3	0	0	0	1	0	2	0	0	0	1	0	0	0	0	0	0	0	0	0	2	0	0	0	0	0	0	0	0	0	0	0	0	0	2	1	1
JHU030	5	0	0	0	2	0	0	0	0	0	0	0	0	0	0	0	0	1	0	2	1	0	0	0	0	0	0	0	0	0	0	0	1	1	2	2	2
JHU031	0	0	0	0	0	0	0	0	0	0	0	0	0	0	0	0	0	0	0	0	0	0	0	0	0	0	0	0	0	0	0	0	0	0	0	0	0
JHU032	5	0	0	0	2	2	2	0	0	0	0	3	2	0	0	0	0	0	0	0	0	0	0	0	1	0	0	0	0	0	0	0	0	0	1	0	0
JHU033	1	0	0	0	2	0	1	1	1	0	1	0	1	0	0	0	1	0	0	0	0	1	1	1	0	0	0	0	0	0	0	0	0	0	0	0	0
JHU034	0	0	0	0	1	0	0	0	1	0	0	1	0	0	0	0	0	0	1	0	0	0	0	0	0	0	0	0	1	0	0	0	1	0	1	0	1
JHU035	1	0	0	0	0	0	1	0	0	0	0	0	0	0	0	0	0	0	0	0	1	0	0	0	0	0	0	0	0	1	0	0	0	2	0	0	0
JHU036	11	0	0	0	2	0	2	2	0	0	0	2	1	0	0	0	0	0	0	2	0	1	1	2	2	0	0	0	0	0	3	2	2	2	0	1	1
JHU037	4	0	0	0	1	1	1	0	1	0	0	2	1	1	1	0	0	0	0	1	1	0	0	0	1	0	0	0	1	0	0	0	0	0	2	2	2
JHU038	10	2	1	2	2	2	1	0	0	0	0	2	2	1	0	0	0	1	1	1	2	1	1	1	1	0	1	0	2	1	0	0	1	3	1	2	0
JHU039	1	0	0	0	0	1	1	0	0	0	0	0	0	0	0	0	0	0	0	0	2	0	0	0	0	0	0	0	0	0	0	0	0	0	0	0	0
JHU040	2	0	0	0	2	0	2	0	0	0	0	1	0	0	0	0	0	0	0	0	0	0	0	0	0	0	0	0	1	0	0	0	0	0	1	1	0
JHU041	0	0	0	0	0	0	0	0	0	0	0	0	0	0	0	0	0	0	0	0	0	0	0	0	0	0	0	0	0	0	0	0	0	0	0	0	0
JHU042	12	0	0	1	3	2	3	0	1	1	1	1	0	3	0	0	0	1	0	0	0	1	3	3	3	2	1	0	0	0	0	0	0	2	2	2	2
JHU043	2	1	0	0	2	1	0	0	1	0	0	2	1	0	0	0	0	0	0	1	1	0	0	0	1	0	0	0	0	0	0	0	0	0	1	1	0
JHU044	6	2	2	1	2	0	0	0	0	0	0	2	0	1	0	0	0	1	0	2	1	0	0	0	0	0	0	1	3	0	0	0	1	0	0	0	0
JHU045	2	0	0	0	2	1	1	0	0	0	0	1	1	1	0	0	0	0	0	0	0	0	2	1	1	0	0	0	0	0	0	0	0	0	0	0	1
JHU046	0	1	0	0	1	1	0	0	1	0	0	0	0	0	0	0	0	0	1	1	0	0	0	0	0	0	0	0	0	0	1	0	0	1	0	0	0
JHU047	0	0	0	0	0	0	0	0	0	0	0	0	0	0	0	0	0	0	0	0	0	0	0	0	0	0	0	0	0	0	0	0	0	0	0	1	0
JHU048	2	0	0	0	0	0	0	0	0	0	0	0	0	0	0	0	0	0	0	2	0	0	0	0	0	0	0	0	0	0	0	0	0	0	2	0	0
JHU049	1	0	0	0	1	1	2	0	0	0	0	0	0	0	0	1	1	0	0	1	1	0	0	0	1	0	0	0	1	0	1	0	0	1	0	0	0
JHU050	1	0	0	0	0	0	0	0	0	0	0	0	0	0	0	0	0	0	0	0	0	0	0	0	1	0	0	0	0	0	0	0	2	0	1	1	0
JHU051	4	0	0	1	2	1	2	0	0	0	0	0	0	0	0	0	0	0	0	2	2	0	1	1	0	0	0	1	0	0	1	0	0	0	0	0	0
JHU052	0	0	0	0	0	0	0	0	0	0	0	0	0	0	0	0	0	0	0	0	1	0	0	0	0	0	0	0	0	0	0	0	0	0	0	0	0
JHU053	1	0	0	0	1	1	1	1	2	0	0	0	0	0	0	0	0	0	1	0	0	0	0	1	1	0	0	0	0	0	0	0	0	0	0	0	0
JHU054	0	0	0	0	0	0	0	0	0	0	0	0	0	0	0	0	0	0	0	0	0	0	0	0	0	0	0	0	0	0	0	0	0	0	0	0	0
JHU055	0	0	0	0	0	0	1	0	0	0	0	1	0	0	0	0	0	0	0	0	0	0	0	0	1	0	0	0	0	0	0	0	0	1	1	0	0

Table 4: Symptoms reported by 6-month Johns Hopkins Biobank patients.

Label	Group	Time point	Fatigue severity scale total score	McGill Pain Questionnaire: McGill total score	McGill affective score	McGill sensory score	Pittsburgh sleep quality index	Beck Depression Index: Beck total score	Beck affective score	Beck sensory score
JHU001	RTH	12 month	21	2	0	2	3	10	8	2
JHU002	PTLD	12 month	11	0	0	0	1	0	0	0
JHU003	PTLD	12 month	45	8	4	4	9	14	8	6
JHU004	RTH	12 month	9	1	0	1	6	1	0	1
JHU005	PTLD	12 month	12	0	0	0	8	10	7	3
JHU006	PTLD	12 month	39	0	0	0	9	15	9	6
JHU007	RTH	12 month	16	0	0	0	3	0	0	0
JHU008	RTH	12 month	23	0	0	0	4	6	4	2
JHU009	RTH	12 month	30	0	0	0	0	0	0	0
JHU010	PTLD	12 month	46	10	1	9	11	10	6	4
JHU011	RTH	12 month	32	3	1	2	6	0	0	0
JHU012	RTH	12 month	31	1	0	1	6	6	2	4
JHU013	RTH	12 month	25	5	0	5	7	3	2	1
JHU014	RTH	12 month	15	0	0	0	2	0	0	0
JHU015	RTH	12 month	16	0	0	0	2	0	0	0
JHU016	RTH	12 month	23	0	0	0	7	2	1	1
JHU017	RTH	12 month	9	1	0	1	1	0	0	0
JHU018	RTH	12 month	18	0	0	0	2	5	4	1
JHU019	PTLD	12 month	23	4	0	4	5	11	5	6
JHU020	RTH	12 month	13	0	0	0	3	1	1	0
JHU021	PTLD	12 month	54	5	0	5	8	20	18	2
JHU022	PTLD	12 month	15	7	2	5	5	2	1	1
JHU023	RTH	12 month	35	1	0	1	4	4	2	2
JHU024	PTLD	12 month	41	7	1	6	7	12	7	5
JHU025	PTLD	12 month	33	0	0	0	9	12	7	5
JHU026	RTH	12 month	9	0	0	0	1	0	0	0
JHU027	RTH	12 month	11	0	0	0	1	0	0	0
JHU028	PTLD	12 month	26	8	0	8	6	15	8	7
JHU029	PTLD	12 month	41	1	0	1	8	11	8	3
JHU030	PTLD	12 month	46	13	4	9	8	9	7	2
JHU031	RTH	12 month	12	0	0	0	0	0	0	0
JHU032	PTLD	12 month	46	12	2	10	5	16	11	5
JHU033	PTLD	12 month	19	3	1	2	6	16	12	4
JHU034	RTH	12 month	9	0	0	0	2	5	3	2
JHU035	RTH	12 month	20	1	0	1	6	3	0	3
JHU036	PTLD	12 month	43	3	1	2	10	1	1	0
JHU037	PTLD	12 month	29	2	0	2	5	15	14	1
JHU038	PTLD	12 month	58	13	2	11	11	16	10	6
JHU039	RTH	12 month	9	0	0	0	3	0	0	0
JHU040	PTLD	12 month	42	2	0	2	3	9	7	2
JHU041	RTH	12 month	33	0	0	0	2	0	0	0
JHU042	PTLD	12 month	45	4	2	2	9	24	17	7
JHU043	PTLD	12 month	40	2	0	2	4	12	7	5
JHU044	PTLD	12 month	63	16	3	13	12	11	3	8
JHU046	RTH	12 month	32	2	0	2	3	7	4	3
JHU047	RTH	12 month	17	1	1	0	2	5	4	1
JHU048	RTH	12 month	16	2	0	2	4	0	0	0
JHU049	PTLD	12 month	41	14	3	11	4	14	8	6
JHU050	RTH	12 month	26	1	0	1	4	10	4	6
JHU051	PTLD	12 month	53	4	1	3	6	7	2	5
JHU052	RTH	12 month	10	11	0	11	3	0	0	0
JHU053	PTLD	12 month	50	4	3	1	7	8	2	6
JHU054	RTH	12 month	17	2	1	1	2	5	3	2
JHU055	RTH	12 month	20	2	0	2	4	4	1	3

Label	SF-36 Health Survey: Physical component score	Mental component score	Mental health score	Role-emotional (RE) score	Societal functioning score	Vitality score	General health score	Bodily pain score	Role-physical (RP) score	Physical functioning score	Post-Lyme Questionnaire of Symptoms: Total score
JHU001	52.75	53.45	55.64	51.99	56.85	48.97	52.93	50.29	54.4	54.93	2
JHU002	59.46	59.44	61.27	55.88	56.85	64.58	63.9	62.12	56.85	57.03	0
JHU003	51.21	37.02	33.11	44.22	45.94	39.6	48.17	46.06	49.51	46.51	5
JHU004	51.56	57.92	55.64	55.88	56.85	61.46	43.4	55.36	56.85	52.82	0
JHU005	62.98	36.19	41.56	36.44	45.94	48.97	57.7	62.12	54.4	52.82	0
JHU006	64.37	30.86	41.56	32.56	35.03	48.97	52.93	62.12	56.85	54.93	6
JHU007	60.99	51.07	50.01	55.88	56.85	52.09	61.51	62.12	56.85	57.03	0
JHU008	61.37	49.12	44.38	55.88	56.85	55.21	57.7	62.12	56.85	57.03	0
JHU009	58.26	55.96	58.46	55.88	56.85	55.21	57.7	62.12	56.85	57.03	0
JHU010	48.22	41.99	44.38	44.22	45.94	39.6	45.78	46.06	42.16	52.82	4
JHU011	56.05	54.56	55.64	55.88	56.85	52.09	55.32	55.36	56.85	57.03	0
JHU012	44.94	56.43	52.82	55.88	56.85	52.09	43.4	41.41	47.06	54.93	2
JHU013	53.68	52.01	50.01	51.99	56.85	52.09	55.32	55.36	49.51	52.82	2
JHU014	61.26	53.27	50.01	55.88	56.85	61.46	61.51	62.12	56.85	57.03	0
JHU015	57.54	53.37	52.82	51.99	56.85	58.33	61.51	50.29	56.85	57.03	0
JHU016	59.39	53.82	55.64	55.88	56.85	52.09	60.08	62.12	56.85	57.03	0
JHU017	57.82	62.35	64.09	55.88	56.85	70.82	59.13	62.12	56.85	57.03	0
JHU018	58.57	52.48	50.01	55.88	56.85	55.21	60.08	55.36	56.85	57.03	0
JHU019	56.24	26.38	30.3	24.78	29.58	52.09	62.47	46.06	34.81	50.72	3
JHU020	57.76	56.74	58.46	55.88	56.85	58.33	55.32	62.12	56.85	57.03	0
JHU021	53.34	22.39	33.11	20.89	29.58	36.48	50.55	55.36	25.02	52.82	10
JHU022	54.2	40.21	41.56	44.22	45.94	45.85	48.17	41.41	56.85	54.93	0
JHU023	51.43	52.75	55.64	51.99	45.94	52.09	56.75	51.13	56.85	46.51	0
JHU024	34.74	47.91	47.19	44.22	45.94	39.6	33.87	37.18	37.26	40.2	20
JHU025	52.87	49.69	52.82	55.88	51.4	36.48	57.7	51.13	54.4	52.82	2
JHU026	59.1	59.46	61.27	55.88	56.85	64.58	62.47	62.12	56.85	57.03	0
JHU027	57.91	59.54	61.27	55.88	56.85	64.58	57.7	62.12	56.85	57.03	0
JHU028	35.72	50.74	50.01	32.56	56.85	52.09	35.3	41.41	32.36	38.09	8
JHU029	52.57	40.82	33.11	55.88	45.94	39.6	55.32	46.06	47.06	52.82	1
JHU030	54.19	32.6	27.48	55.88	35.03	33.36	45.78	41.41	56.85	54.93	6
JHU031	57.64	57.01	55.64	55.88	56.85	61.46	59.13	62.12	56.85	52.82	0
JHU032	45.79	42.8	44.38	44.22	35.03	48.97	52.93	37.18	39.71	50.72	8
JHU033	48.57	47.98	52.82	44.22	45.94	45.85	52.93	41.83	56.85	44.41	0
JHU034	59.47	49.83	47.19	55.88	56.85	52.09	52.93	62.12	56.85	57.03	0
JHU035	53.67	57.84	58.46	55.88	56.85	58.33	52.93	51.13	56.85	57.03	1
JHU036	50.95	53.35	58.46	55.88	51.4	42.72	50.55	51.13	51.96	57.03	2
JHU037	57.8	22.17	24.66	24.78	40.49	39.6	48.17	46.06	44.61	52.82	9
JHU038	45.95	31.42	33.11	40.33	35.03	30.24	37.68	41.41	44.61	46.51	12
JHU039	58.49	54.77	52.82	55.88	56.85	61.46	52.93	62.12	56.85	57.03	0
JHU040	42.32	41.38	41.56	36.44	45.94	42.72	45.78	37.18	37.26	44.41	3
JHU041	56.58	54.69	58.46	51.99	51.4	61.46	50.55	62.12	54.4	57.03	0
JHU042	58.12	21.92	24.66	32.56	35.03	33.36	45.78	46.06	54.4	50.72	11
JHU043	48.47	43.37	52.82	40.33	40.49	42.72	45.78	55.36	37.26	52.82	2
JHU044	20.8	48.66	50.01	55.88	13.22	30.24	36.25	41.83	17.67	25.47	7
JHU046	53.42	50.09	50.01	51.99	56.85	45.85	52.93	46.06	56.85	54.93	1
JHU047	64.01	39.03	35.93	44.22	45.94	61.46	54.36	62.12	56.85	54.93	0
JHU048	57.81	54.08	52.82	55.88	56.85	58.33	50.55	62.12	56.85	57.03	0
JHU049	37.01	40.74	41.56	40.33	35.03	42.72	35.3	41.41	22.57	48.61	7
JHU050	44.5	50.15	50.01	51.99	51.4	42.72	35.3	51.13	44.61	50.72	0
JHU051	31.87	50.37	52.82	44.22	35.03	45.85	43.4	37.18	22.57	42.3	5
JHU052	47.73	64.03	64.09	55.88	56.85	64.58	50.55	46.06	56.85	50.72	1
JHU053	21.83	43.19	47.19	44.22	18.67	27.11	30.53	32.96	27.47	23.36	2
JHU054	53.15	54.36	58.46	51.99	56.85	48.97	50.55	55.36	54.4	54.93	0
JHU055	50.5	56.93	55.64	51.99	56.85	58.33	57.7	41.41	54.4	52.82	1

Label	Post-Lyme Questionnaire of Symptoms (0=absent, 1=mild, 2=moderate, 3=severe): Fever	Chills	Sweats	Fatigue	Muscle pain	Joint pain	Joint swelling	Numbness in hands/feet	Numbness in face and scalp	Muscle twitching	Headache	Eyes sensitive to light	Vision clarity	Double vision	Dropping face	Drooping eyelid	Dizziness	Ringing in ears
JHU001	0	0	1	1	1	1	0	2	0	0	2	0	0	0	0	0	0	1
JHU002	0	0	0	0	0	0	0	0	0	0	0	0	0	0	0	0	0	0
JHU003	1	0	0	3	1	3	1	1	0	0	1	0	0	0	0	0	0	0
JHU004	0	0	1	1	1	1	0	1	0	0	1	0	0	0	0	0	0	0
JHU005	0	0	0	0	0	0	0	0	0	0	0	0	0	0	0	0	0	0
JHU006	0	0	0	2	0	1	0	1	0	0	0	0	2	0	0	0	0	0
JHU007	0	0	0	1	0	0	0	0	0	0	1	0	0	0	0	0	0	0
JHU008	0	0	0	0	0	0	0	0	0	0	0	0	0	0	0	0	0	0
JHU009	0	0	0	1	0	0	0	0	0	0	1	0	0	0	0	0	0	0
JHU010	0	0	0	1	1	2	1	2	0	1	2	0	1	0	0	0	0	0
JHU011	0	0	0	1	0	0	0	0	1	1	1	0	0	0	0	0	0	0
JHU012	1	1	1	1	1	1	1	1	1	1	0	0	0	0	0	0	0	0
JHU013	0	0	0	1	1	1	0	0	0	0	1	0	0	0	0	0	1	0
JHU014	0	0	0	0	0	0	0	0	0	0	0	0	0	0	0	0	0	0
JHU015	0	0	0	1	0	0	0	0	0	1	0	0	0	0	0	0	0	0
JHU016	0	0	0	0	0	0	0	0	0	0	0	0	0	0	0	0	0	0
JHU017	0	0	0	0	0	0	0	0	0	0	1	0	0	0	0	0	0	0
JHU018	0	0	0	1	1	1	0	0	0	0	1	0	0	0	0	0	0	0
JHU019	0	0	1	0	0	2	0	0	0	0	0	1	0	0	0	0	0	0
JHU020	0	0	0	0	0	0	0	0	0	0	0	0	0	0	0	0	0	0
JHU021	0	0	0	2	2	2	0	2	0	2	1	1	0	0	0	0	0	1
JHU022	0	0	0	1	1	0	0	0	0	0	0	1	0	0	0	0	0	0
JHU023	0	0	1	0	1	1	1	0	0	0	0	1	0	0	0	0	0	0
JHU024	1	2	2	3	1	2	2	2	1	0	2	2	2	0	0	0	1	0
JHU025	0	0	0	2	1	2	1	0	0	0	1	1	0	0	0	0	0	0
JHU026	0	0	0	0	0	0	0	0	0	0	0	0	0	0	0	0	0	1
JHU027	0	0	0	0	0	0	0	0	0	0	0	0	0	0	0	0	0	0
JHU028	0	0	0	1	0	2	2	2	0	0	2	1	0	0	0	0	0	1
JHU029	0	0	0	1	1	1	0	0	0	0	1	0	0	0	0	0	0	0
JHU030	0	0	0	2	0	0	0	0	0	0	0	0	0	0	0	0	0	0
JHU031	0	0	0	0	0	0	0	0	0	0	0	0	0	0	0	0	0	0
JHU032	0	0	0	2	1	3	0	2	0	1	1	1	2	0	0	0	0	0
JHU033	0	0	0	1	0	1	1	1	0	0	1	1	0	0	0	1	0	0
JHU034	0	0	0	1	0	0	0	0	0	0	1	0	0	0	0	0	1	1
JHU035	0	0	0	0	0	0	0	0	0	0	0	0	0	0	0	0	0	0
JHU036	0	0	0	2	0	1	0	0	0	0	1	1	0	0	0	0	0	0
JHU037	1	0	1	1	2	0	0	0	0	1	2	1	2	0	0	0	1	0
JHU038	0	0	0	2	0	2	0	0	0	1	2	2	0	0	0	0	0	1
JHU039	0	0	1	0	0	0	0	0	0	0	1	1	0	0	0	0	0	0
JHU040	1	1	0	2	1	2	0	0	0	1	0	0	1	0	0	0	1	0
JHU041	0	0	0	0	0	0	0	0	0	0	0	0	0	0	0	0	0	0
JHU042	0	1	1	3	0	2	0	0	0	0	2	1	3	0	0	0	1	0
JHU043	0	0	1	2	1	0	0	0	0	0	2	1	1	0	0	0	0	0
JHU044	1	1	1	3	0	0	0	0	0	0	1	0	2	0	0	0	3	0
JHU046	0	0	0	0	0	1	0	2	0	0	0	0	0	0	0	0	0	0
JHU047	0	0	0	0	0	0	0	0	0	0	0	0	0	0	0	0	0	0
JHU048	0	0	0	0	0	0	0	0	0	0	0	0	0	0	0	0	0	0
JHU049	0	0	0	1	1	2	1	0	0	0	2	1	0	0	1	1	1	0
JHU050	0	0	1	0	0	1	0	0	0	0	0	1	1	0	0	0	0	0
JHU051	0	0	0	2	0	1	0	0	0	0	1	1	0	0	0	0	1	0
JHU052	0	0	0	0	0	0	0	0	0	0	1	0	0	0	0	0	0	0
JHU053	0	0	0	3	1	1	1	1	0	0	0	1	0	0	0	0	1	1
JHU054	0	0	0	1	1	1	0	0	0	0	0	0	0	0	0	0	0	0
JHU055	0	0	0	1	1	0	0	0	0	0	1	0	0	0	0	0	0	0

Label	Neck pain	Lower back pain	Poor coordination	Memory impairment	Difficulty winding words	Difficulty focusing or concentrating	Heart palpitations	Difficulty breathing	Swollen lymph nodes	Sore throat	Urination changes	Nausea	Vomiting	Diarrhea	Sleep disruption	Anxiety	Depression	Irritability
JHU001	1	0	0	0	1	0	0	0	0	0	0	0	0	0	1	0	0	0
JHU002	0	0	0	0	0	0	0	0	0	0	0	0	0	0	0	0	0	0
JHU003	0	2	0	1	1	1	0	0	0	1	0	0	0	1	1	2	1	2
JHU004	1	1	0	0	0	0	0	0	0	0	0	0	0	0	1	0	0	0
JHU005	0	0	0	0	0	0	0	0	0	0	0	0	0	0	1	1	0	0
JHU006	0	3	1	1	2	2	0	0	0	0	0	0	0	0	2	0	1	1
JHU007	0	0	0	0	0	0	0	0	0	0	0	0	0	0	0	0	0	0
JHU008	0	0	0	0	0	0	0	0	0	0	0	0	0	0	0	1	1	1
JHU009	0	0	0	0	0	0	0	0	0	0	0	0	0	0	0	0	0	0
JHU010	0	1	1	2	1	1	0	0	0	0	0	0	0	0	0	0	0	0
JHU011	0	0	0	1	1	0	0	0	0	0	0	0	0	1	1	1	0	0
JHU012	1	0	0	0	0	0	2	0	1	0	0	0	0	1	2	1	1	1
JHU013	0	0	0	0	1	1	1	0	0	2	3	0	0	1	1	1	1	0
JHU014	0	0	0	0	0	0	0	0	0	0	0	0	0	0	0	0	0	0
JHU015	0	0	0	0	0	0	0	0	0	0	0	0	0	0	1	0	0	0
JHU016	0	0	0	0	0	0	0	0	0	0	0	0	0	0	1	0	1	0
JHU017	0	1	0	0	0	0	0	0	0	0	0	0	0	0	0	0	0	0
JHU018	0	1	0	0	0	0	0	0	0	0	0	0	0	0	0	1	0	1
JHU019	0	1	0	2	1	1	0	0	0	0	0	0	0	0	0	2	0	0
JHU020	0	0	0	0	0	0	0	0	0	0	0	0	0	0	0	0	0	0
JHU021	1	0	1	0	1	1	2	1	0	1	0	0	0	0	2	2	2	2
JHU022	1	1	0	0	0	0	0	0	0	0	0	0	0	0	0	1	1	1
JHU023	0	1	0	1	1	0	1	0	0	0	0	0	0	0	0	1	0	0
JHU024	2	2	2	2	2	2	0	0	0	0	0	2	0	2	2	1	2	2
JHU025	0	1	0	1	1	1	0	1	0	0	0	0	0	0	0	1	0	1
JHU026	0	0	0	0	0	0	0	0	0	0	0	0	0	0	0	0	0	0
JHU027	0	0	0	0	0	0	0	0	0	0	0	0	0	0	0	0	0	0
JHU028	2	2	1	1	2	1	1	0	0	0	0	1	0	2	0	1	1	0
JHU029	0	1	0	0	0	0	0	0	0	0	0	0	0	0	0	2	1	0
JHU030	2	1	0	0	0	0	0	0	0	1	0	0	0	0	2	2	2	2
JHU031	0	0	0	0	0	0	1	0	0	0	0	0	0	0	0	0	0	0
JHU032	0	0	1	2	2	2	1	0	0	0	0	0	0	0	2	1	0	1
JHU033	1	0	0	1	1	0	0	0	0	1	1	0	0	0	0	0	0	1
JHU034	0	0	0	0	0	0	0	0	0	0	0	0	0	1	0	1	0	1
JHU035	0	2	0	0	0	0	0	0	0	0	1	0	0	0	1	0	0	0
JHU036	0	1	1	0	1	2	0	0	0	0	0	0	0	0	1	1	0	0
JHU037	1	1	1	1	1	2	0	0	1	1	0	0	0	2	2	2	2	2
JHU038	2	2	1	2	2	2	0	0	0	1	0	0	0	0	3	2	2	1
JHU039	0	1	0	0	0	0	0	0	0	0	0	1	0	0	0	0	0	0
JHU040	1	1	1	0	0	1	0	0	0	1	0	0	0	1	2	1	1	1
JHU041	0	0	0	0	0	0	0	0	0	0	0	0	0	0	0	0	0	0
JHU042	0	0	1	3	3	3	0	1	0	0	0	0	0	0	2	3	2	3
JHU043	0	0	0	0	1	1	0	0	0	1	0	1	0	0	1	1	1	1
JHU044	0	0	1	1	1	1	2	0	0	0	1	3	1	2	2	0	0	0
JHU046	1	0	0	0	0	0	0	0	0	0	0	0	0	0	0	0	0	0
JHU047	0	0	0	0	0	0	0	0	0	0	0	0	0	0	0	1	1	0
JHU048	1	0	0	0	0	0	0	0	0	0	0	0	0	0	0	0	0	0
JHU049	2	2	1	1	1	1	1	0	0	1	0	0	0	0	2	3	1	2
JHU050	0	0	0	0	1	0	0	0	0	0	0	0	0	0	0	0	0	0
JHU051	2	0	0	2	2	2	0	0	0	0	0	0	0	0	0	1	1	1
JHU052	3	1	0	0	0	0	0	0	0	1	0	0	0	0	0	0	0	0
JHU053	0	0	0	1	1	1	1	1	0	2	0	0	0	1	0	0	1	0
JHU054	0	0	0	1	1	0	0	0	0	0	0	0	0	0	0	0	0	0
JHU055	0	2	0	0	0	1	0	0	0	0	0	0	0	0	0	1	1	1

Table 5: Symptoms reported by 12-month Johns Hopkins Biobank patients.
